# Optoelectronic polymer memristors with dynamic control for power-efficient in-sensor edge computing

**DOI:** 10.1038/s41377-025-01986-9

**Published:** 2025-09-08

**Authors:** Jia Zhou, Wen Li, Ye Chen, Haowen Qian, Yen-Hung Lin, Ruipeng Li, Zhen Wang, Jin Wang, Wei Shi, Xianwang Tao, Youtian Tao, Haifeng Ling, Wei Huang, Mingdong Yi

**Affiliations:** 1 State Key Laboratory of Flexible Electronics, Institute of Advanced Materials (IAM), Nanjing University of Posts & Telecommunications (NUPT), Nanjing, 210023 China; 2 Department of Electronic and Computer Engineering, The Hong Kong University of Science and Technology, Clear Water Bay, Kowloon, Hong Kong SAR, China; 3 State Key Laboratory of Advanced Displays and Optoelectronics Technologies, The Hong Kong University of Science and Technology, Clear Water Bay, Kowloon, Hong Kong SAR, China; 4 National Synchrotron Light Source II, Brookhaven National Laboratory, Upton, NY 11973 USA; 5 Key Lab for Flexible Electronics and Institute of Advanced Materials, Nanjing Tech University, Nanjing, 211816 China

**Keywords:** Optical data storage, Photonic devices

## Abstract

As the demand for edge platforms in artificial intelligence increases, including mobile devices and security applications, the surge in data influx into edge devices often triggers interference and suboptimal decision-making. There is a pressing need for solutions emphasizing low power consumption and cost-effectiveness. In-sensor computing systems employing memristors face challenges in optimizing energy efficiency and streamlining manufacturing due to the necessity for multiple physical processing components. Here, we introduce low-power organic optoelectronic memristors with synergistic optical and mV-level electrical tunable operation for a dynamic “control-on-demand” architecture. Integrating signal sensing, featuring, and processing within the same memristors enables the realization of each in-sensor analogue reservoir computing module, and minimizes circuit integration complexity. The system achieves 97.15% fingerprint recognition accuracy while maintaining a minimal reservoir size and ultra-low energy consumption. Furthermore, we leverage wafer-scale solution techniques and flexible substrates for optimal memristor fabrication. By centralizing core functionalities on the same in-sensor platform, we propose a resilient and adaptable framework for energy-efficient and economical edge computing.

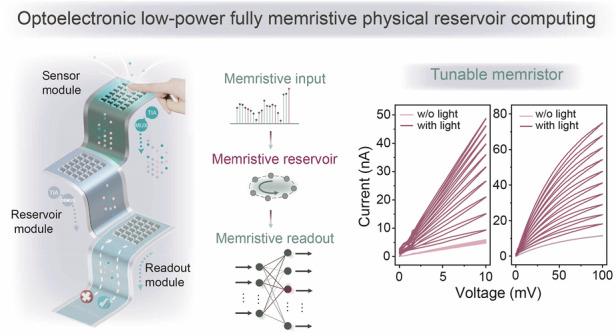

## Abstract (web summary)

Tunable mV-level optoelectronic polymer memristors enabling ultra-low power in-sensor reservoir computing for highly accurate and efficient edge computing applications.

## Introduction

Memristor arrays usually constitute neural networks with in-memory computing and in-sensor computing technology, allowing real-time, on-location signal processing with reduced data movement^1,2^. However, the inherent structure of fully connected network requires storing and updating the weights of thousands of nodes, leading to a significant increase in energy consumption and reduced area efficiency^3^. In edge computing, frequent data conversions through analogue-to-digital converters (ADCs) and large volumes of weight data transfer among physically separated units demand energy-efficient and compact form factor devices to manage their computing resources effectively and operate with high accuracy^4–8^. Considering these constraints, memristor-based computing systems suffer from drawbacks that significantly restrict their practical utility, including large fully connected layer sizes^9^, high voltage overheads for memristor writing^10,11^ and additional ADCs^1,12^. In contrast, memristor-based in-sensor physical reservoir computing (RC) systems have the potential for small-size training networks^13^. The reservoir units extract additional temporal features from the analogue input data stream through nonlinear transformation^14–16^, achieving low-cost RC by limiting the training process to the high-dimensional weights connecting to the linear readout layer^17–19^.

Combining different memristive device specifications to meet the unique requirements of the reservoir layer and read-out layer ultimately increases the complexity of the RC frameworks^20,21^. Moreover, most filament-based memristors exhibit stochastic electroforming steps and binary conductance states, which usually require high operation voltages to activate networks, as well as additional reading processes^6,10,11,22,23^. This limitation hinders direct signal processing in the original analogue in-sensor memristive form, particularly in real-world applications such as conventional security and personal identification^24^. Memristors can create multimode neuromorphic operations that switch as needed, provided that the spatiotemporal dependence of the optical dimensions is harnessed effectively^25–27^. Although low-power optoelectronic organic memristors have been developed to simplify various recognition tasks through RC, most of them rely on heterojunction-based functional layers, which limit the scalability of device fabrication over large areas and fall short of supporting reconfigurable functionalities for both reservoir and read-out layers within a single device. Organic polymers are well-suited for constructing such in-sensor systems^28^, their low thermal budget and low-cost solution-based fabrication process, which allow precise tuning of intermolecular stacking and crystallinity in polymer films, have the potential to facilitate efficient charge transport thereby enabling rich and broad configurable photoelectric responsive tunability^2,29–31^. Together with intrinsic flexibility, these are valuable attributes for enhancing the layout of multifunctional low-power memristors to simplify the in-sensor system at the hardware level^4,21^.

The continuous advance of edge computing underscores the necessity to construct analogue in-sensor nodes using ultralow-power electronics, combined with reconfigurable dynamics and promising architectures^32–36^. In this study, we introduce forming-free optoelectronic organic polymer memristors, demonstrating multiple photoconductance states adjustable via ultra-low voltages. The polymer is manufacturable using a scalable solution process on flexible substrates, ensuring cost-effectiveness. Building upon the achievement of sensing functionality, simple voltage adjustments enable the configuration of nonlinear and linear regions for low-power RC operations, all within a single type of memristor. This innovative, space-efficient in-sensor architecture supports diverse computational primitives without additional ADCs or data movements. Taking the fingerprint recognition task as an example, the system achieves a fingerprint recognition accuracy of 97.15% with a compact reservoir architecture and minimal energy usage. Edge extraction in the optoelectronic organic memristor array proves more economical than conventional user interfaces, potentially hastening practical applications in future advanced intelligent systems.

## Results

### Multi-functional optoelectronic memristive features

**Fig. 1:**
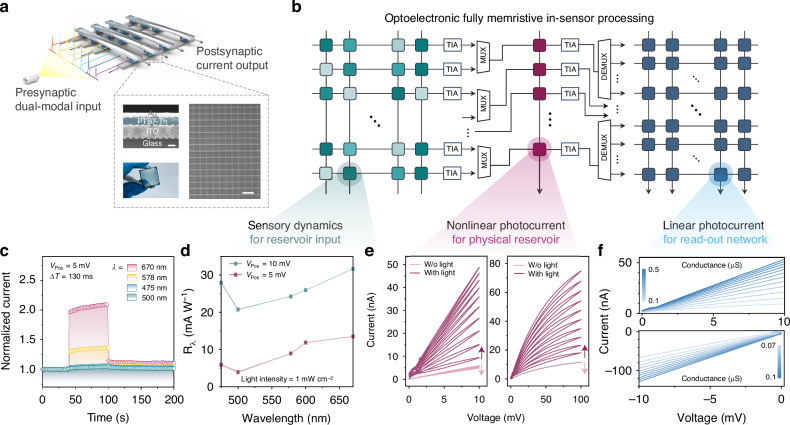
**Reconfigurable optoelectronic memristors for versatile neuromorphic computing.** **a** Schematic diagram of the crossbar array structure with optoelectronic memristors (top), and cross-sectional (scale bar: 100 nm), top view (scale bar: 500 μm) SEM image, and optical image of the fabricated multifunctional memristor (bottom). **b** Schematic of the analogue optoelectronic fully memristive in-sensor RC system. **c** Broadband optoelectronic memristive characteristics and **d** Photoresponsivity under the same light intensity (1 mW cm^–2^). **e** Linear *I–V* characteristics at low driving voltages enable direct analogue input to the device, while enhanced nonlinearity at higher voltages supports reservoir module implementation. The device exhibits counterclockwise hysteresis under illumination and clockwise hysteresis in darkness. **f** Linear *I–V* characteristics at different conductance states

To counter these challenges, we built a compact RC system for future in-sensor platforms using the same type of tunable organic optoelectronic memristors. We introduced the poly([2,6’-4,8-di(5-ethylhexylthienyl)benzo[1,2-b;3,3-b]dithio-phene]{3-fluoro-2[(2-ethylhexyl)carbonyl]t-h-ieno[3,4-b]thiophenediyl}) (PTB7-Th) via solution processing to form functional layers at each crossbar intersection. The vertically stacked cross-point structure with the optoelectronic PTB7-Th memristive cell is schematically described in the top panel of Fig. 1a. We fabricated a 20 × 20 array with well-separated layers confirmed by both cross-sectional and top-view scanning electron microscopy (SEM) images. The memristor provides tunable parameters for tailoring optoelectronic behaviours to realise specific device functionalities as depicted in Fig. 1b. In the proposed in-sensor, fully memristive processing system, a single type of PTB7-Th memristor simultaneously performs sensing, nonlinear mapping, and readout. Reflected light produces spatially varying photocurrents in the sensor layer, which are subsequently converted into voltage signals by trans-impedance amplifiers (TIAs). These signals are then routed through a multiplexer (MUX) into the reservoir layer. The analogue dynamics within the reservoir project the inputs into a high-dimensional state space, enabling the memristive readout layer to complete classification, all conducted entirely in the analogue domain without digitisation. In the present work, unless otherwise stated, all voltage stimuli were applied to the ITO electrodes while grounding the metal electrodes. Figure 1c demonstrates broadband signal sensing operations at different wavelengths. The spectral photoresponse of the PTB7-Th device was characterised under applied biases of 5 and 10 mV, with illumination wavelengths ranging from 320 to 670 nm (Fig. 1d). A pronounced responsivity peak was observed at 670 nm, gradually decreasing toward shorter wavelengths, which is consistent with the absorption onset of the polymer. The wavelength-dependent photoresponses enable enhanced spectral selectivity and multi-channel encoding, providing a richer feature space for high-dimensional optoelectronic computing tasks. The linear current–voltage (*I*–*V*) curves of the memristor under low-voltage conditions are shown in the left panel of Fig. 1e. As the voltage amplitude increases, the *I–V* characteristics transition from linear to nonlinear (right panel). Under dark conditions, both linear and nonlinear voltage sweeps result in a gradual decrease in current over successive cycles, indicating continuous increase in device resistance. In contrast, under illumination, the current progressively increases with each voltage sweep, reflecting an analogue and multilevel transition towards a low-resistance state. By incorporating temporal input history and increased voltage amplitudes under light conditions, the analogue nonlinear relationship between conductivity and external optical or electrical stimuli shows promise as a feature space for optoelectronic reservoir mapping. Utilising linear weight updates at low voltages, Fig. 1f illustrates the bidirectional mV-level electric programmability without an initial high-energy activation step and allowing for modulating the ultralow operating currents for multiple conductive states. The linear characteristics ensure consistent resistance states under different input voltage levels. This enables the direct processing of analogue signals as inputs for the readout module without utilising ADCs. The coordinated regulation of operating voltage and light illumination acts as a switch for converting between nonlinear (physical reservoir module) mode and linear (readout module) mode, offering a promising “control-when-required” approach to simplifying integrated circuits and reducing power consumption.

### Mechanism of (non)linear updating memristive states

**Fig. 2:**
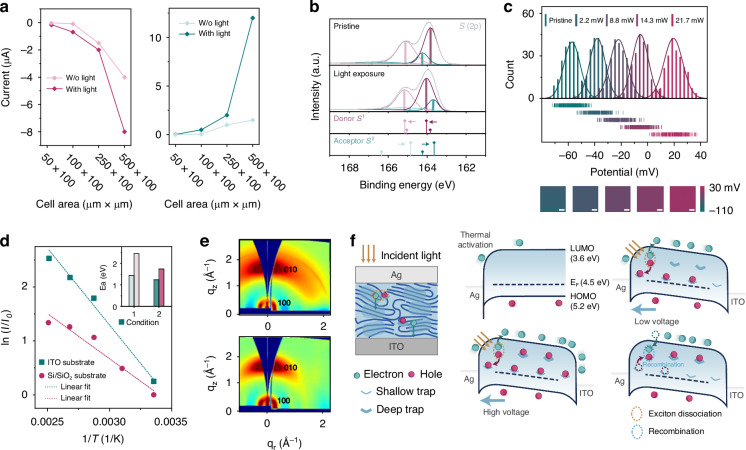
**Mechanisms underlying tunable memristive operations.** **a** Cell-area-dependent memristive characteristics. The current increase under both negative (left) and positive (right) biases with the increase in device area, excluding the possibility of a filamentary switching mechanism. The current values at 0.1 V (−0.1 V) were extracted from *I–V* sweeps ranging from 0 to 0.1 V (−0.1 V) for devices with varying cell areas. Light intensity: 5 mW cm^–2^. **b** XPS spectra of the S (2p) states for PTB7-Th films before and after 1 h of light exposure. **c** The surface potential values (top) and the corresponding KPFM images (bottom) under various intensities of light (scale bar: 250 nm). **d** Photocurrent as a function of temperature and variation of *Ea* at increasing temperature (inset), which depends on the Arrhenius equation: *I* = *I*_0_ × exp(−*Ea*/k*T*). Condition 1: 100 °C, condition 2: 125 °C. **e** Structural properties analysed by GIWAXS, with 2D patterns of PFB7-Th (70 kDa) film on ITO substrate (top) and Si/SiO_2_ substate (bottom). **f** Schematic illustration of the photoinduced charge transport and trapping mechanisms in PTB7-Th memristors under low and high voltage conditions

To demonstrate the tunable concept outlined in Fig. 1, we conducted a systematic mechanistic study on the origin of memristive phenomena. Figure 2a shows that the PTB7-Th devices exhibit homogeneous optoelectronic memristive behaviour on micrometre scales, with photocurrent increasing proportionally to the device area. The significant dependence of resistance on device area indicates that switching is governed by spatially uniform interfaces and a non-filamentary process. Downscaling the PTB7-Th memristors to nanometre scale maintains spatial homogeneity of the internal electric field and stable optoelectronic properties (Fig. S1). Figures S2–S4 provide a control supplement of the active layer thickness, electrode effect, and device structure to identify the intrinsic roles of the PTB7-Th film. In addition, devices that were not exposed to ambient air performed the same characteristics in a vacuum (Fig. S5), indicating that ambient exposure to oxygen and moisture does not affect the optoelectronic memristive phenomena. Under visible-light irradiation, the donor-acceptor type polymer undergoes intramolecular electron transfer^37^, as revealed by X-ray photoelectron spectroscopy (XPS) analysis. Figure 2b shows changes in S (2p) core-level spectra of PTB7-Th films under dark and light conditions. After light exposure, the S^1^ 2p peak in the donor units shifts to higher binding energy, while the S^2^ 2p peak in the acceptor units shifts in the opposite direction, indicating electron transfer from donor to acceptor units^38^. Figures S6–S9 further demonstrate efficient exciton dissociation in the film. Kelvin probe force microscopy (KPFM) confirms that light exposure generates positive charges, with the contact potential difference between PTB7-Th and the KPFM tip decreasing under higher light intensities, indicating increased trapped holes, as shown in Fig. 2c^39^. Holes localise rapidly upon generation, while the majority of mobile carriers are electrons, contributing to the increase in photocurrent. The combination of low initial carrier states, sufficient photogenerated carriers to fill traps, the photogating effect, and the persistent photoconductivity (PPC) endow the PTB7-Th memristor with excellent linearity in weight updating when subjected to lower voltage inputs. As applied voltage increases, the electric field induces the accumulation of electrons in the film, accelerating recombination with holes in shallow traps, thereby reducing the photoconductivity update rate. The capture of carriers by deep traps is also promoted, enabling nonlinear weight updating. Upon switching off the light, the photocurrent decreases rapidly due to electron-hole recombination within shallow traps. Subsequently, this recombination becomes limited, primarily attributed to holes in deeper traps. This is also confirmed by the KPFM measurement of the PTB7-Th film surface potential before and after applying the optical spikes in Fig. S10.

In the case of PTB7-Th memristors, we correlate nonlinear performance with deep traps and the Arrhenius-type activation energy (*Ea*)^40^. The low measured *Ea* for the film on the silicon substrate reflects fewer deep traps in the active layer, as shown in Fig. 2d. The *Ea* exhibits a negative correlation with high temperature in the inset, consistent with increased *I–V* linearity in Fig. S11. The charge transport process occurs after the injected charges fill the traps. Once the trap states are sufficiently filled, the excess injected carriers gain sufficient thermal energy to overcome the potential barrier at the electrode and semiconductor interface. The temperature dependence of charge transport reflects the *Ea* required for carriers to escape from trap states into mobile transport levels. Here, the process can be described by the thermionic emission model^13^, where a linear relationship between ln(*I*/*I*_*0*_) and *V*^1/4^ is observed, confirming the thermionic-assisted carrier injection under illumination. Figures S12–S15 and Note S1 provide a detailed and complete analysis. The photocurrent decay after light exposure follows a time-dependent bi-exponential decay, implying a fast mechanism caused by intrinsic photoconduction and a slow decay mechanism caused by deep traps. We utilised Grazing-Incidence Wide-Angle X-ray Scattering (GIWAXS) to gain deep insights into the ordering of PTB7-Th films, as shown in Fig. 2e. The layered diffraction peak (100) of PTB7-Th exhibits greater intensity in the in-plane direction, whereas the strong (010) peak corresponding to π-π stacking in the out-of-plane direction^41^, indicating face-to-face polymer stacking enhances charge transmission to the electrode^42^. The relative disorder in the in-plane diffraction pattern indicates more potential trapping sites, facilitating the dissociation of the excitons into free charges, thereby increasing the probability of charge trapping^43,44^. We speculate that the defects caused by the moderate structural disorder of the film optimise memristive performance, providing required linearity and nonlinearity. This is confirmed by the film microstructure and memristive performance of the fabricated devices on different substrates. Films deposited on silicon substrates show a more intense out-of-plane π-π stacking diffraction arc, reducing energy disorder and defect states. Figure S16 shows clear linear conductance switching in the *I–V* curves of the silicon substrate device. However, PTB7-Th films with different molecular weights show negligible differences regarding the molecular orientation or stacking modes. The low molecular weight polymer (50 kDa) exhibits a broader arc-like scattering pattern, indicating more disorder, which promotes trapping during charge transport processes and induces more recombination losses, negatively affecting memristive performance stability. We propose that the combined effects of efficient exciton dissociation induced by intramolecular charge transfer in the PTB7-Th polymer, through-space charge transport facilitated by its vertical stacking orientation, and carrier trapping regulated by trap-state dynamics enable the device to exhibit tunable photoresponsive synaptic behaviours and nonlinearly separable fading memory under different light conditions^31^. Figure 2f schematically illustrates the photophysical and trapping processes that govern the optoelectronic memristive behaviour of the PTB7-Th devices under different bias conditions. Upon photoexcitation, excitons dissociate into mobile electrons and holes, with most holes becoming rapidly trapped. The in-plane disorder of the polymer favours hole trapping, while the out-of-plane π–π stacking facilitates efficient vertical electron conduction. At low voltage bias, the photogenerated carriers fill shallow traps, sustaining the photogating and PPC effects that contribute to linear synaptic weight modulation. As the applied voltage increases, the electric field enhances electron accumulation and accelerates recombination with holes in shallow traps, thereby reducing the photocurrent. Simultaneously, it enables the capture of carriers by deeper traps, introducing nonlinearity into the conductance response. After the light is turned off, a rapid decay in photocurrent is observed due to fast recombination in shallow traps, followed by a slower relaxation phase governed by deeply trapped holes. These deep traps retain charges over extended timescales and provide the basis for fading memory, allowing the device to encode the temporal history of optical stimuli. These characteristics provide a solid foundation for implementing RC functions within a single memristive device.

### Neuromorphic characteristics of optoelectronic memristors

**Fig. 3:**
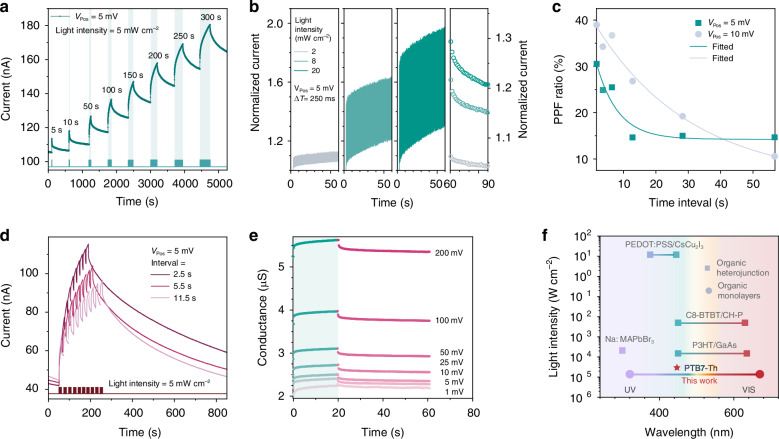
**Low-voltage optoelectrical synaptic behaviours.** Progressive multi-state photoconductivity demonstrates stable synaptic weight potentiation and PPC effect under **a** different durations and **b** different light intensities for in-sensor computing. **c** PPF ratio as a function of the optical pulse interval where paired optical pulses were applied under 5 or 10 mV bias. The experimental data are fitted using the double-exponential decay function: PPF index = 1 + *C*_1_exp(−Δ*t*/*τ*_1_) + *C*_2_exp(−Δ*t*/*τ*_2_). **d** The prominent accumulation effect induced by a train of optical pulses with multiple frequencies, showing temporal correlations. **e** The conductance change stimulated by different electrical biases (1, 5, 10, 25, 50, 100, and 200 mV) with (0–20 s) and without (20–60 s) light illumination (all use 5 mW cm^–2^ white light sources). **f** Comparison of previously reported organic two-terminal optoelectronic memristors as the reservoir module in terms of wavelength and intensity of light stimulus (see details in Table S1)

A fundamental understanding of the photoresponse characteristics of individual memristors is crucial for designing various in-sensor computational functions at the device level. Figure 3a shows the stepwise increase in photocurrent under constant light intensity for eight different durations. The results indicate a direct correlation between the photocurrent and exposure duration, with each state demonstrating good stability. As input voltage increases, the background carriers gradually screen the effect of photogenerated carriers, leading to a low-magnitude weight update. Figure 3b illustrates the progressive multistate photocurrent under constant voltage (5 mV), tuned with varying light intensities. Memristors can convert optical patterns with varying levels of brightness into a series of analogue photocurrents, where higher light intensity results in prolonged retention time. Decreasing the light pulse interval significantly enhances synaptic strength. Figure 3c demonstrates the enhancement of the second spike amplitude of two evoked excitatory postsynaptic currents, analogous to paired-pulse facilitation (PPF) in biology, which is essential for memristors to correlate the temporal spike pairs. We verified the preservation of temporal correlation by examining diverse light pulse intervals (Fig. 3d) and assessing the excitatory postsynaptic current (EPSC) response alongside fading memory over up to ten light pulses. The analogue-type photoconductance can also be gradually and continuously controlled by the driven voltage, allowing individual tuning of the sensitivity of each cell in the array, as characterised in Fig. 3e. Furthermore, recognition under broadband light and dim light can be challenging for memristive architecture. Figure S17 shows the dependence of the memristive photocurrent on light intensity at multiple wavelengths. Figures S18 and S19 additionally shows the light-induced EPSC behaviour under dim light of different wavelengths. These results still match comparable organic memristors for optoelectronic reservoir layers at a competitive level (Fig. 3f, see Table S1 for full comparisons). Notably, the process also enables large-area semiconductor technology compatible with the Silicon platforms in Fig. S20 and flexible electronics in Fig. S21. The retention of low-voltage memristive functions after bending cycles indicates their suitability for future portable flexible edge applications.

### Optoelectronic RC system and design rules

**Fig. 4:**
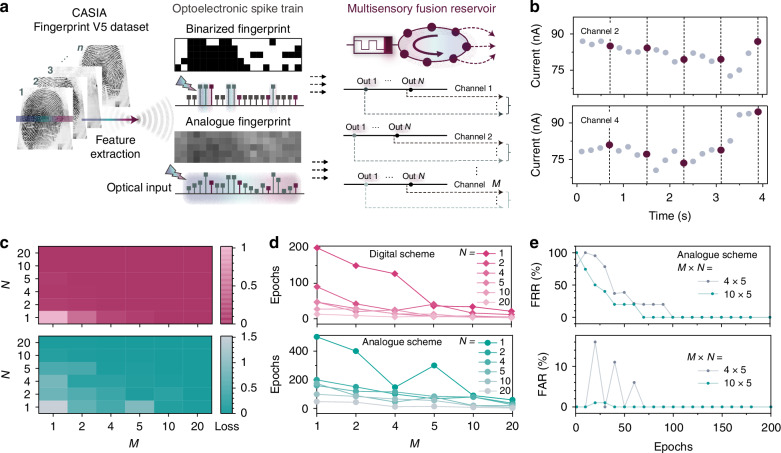
**RC system architecture and adjustments for improving reservoir performance.** **a** The schematic of the feature extraction from different inputs for the memristor-based RC system. **b** Temporal response of the memristor current (grey dots) to the spike trains in two representative channels. The complete input can be divided into different intervals, representing different virtual nodes (burgundy dots) whose states are measured at the end of the intervals. **c** The loss during 200 epochs of the training process changes with the number of physical nodes *M* and virtual nodes *N* in the digital scheme (top) and analogue scheme (bottom). **d** The number of epochs during the training process when the classification accuracy reaches 100% for the first time remains stable in the digital and analogue schemes. **e** The evolution of the *FRR* and *FAR* during the training process

Memristors, within the RC paradigm, function as reservoirs with their intrinsic short-term memory and nonlinear internal dynamics. They project input signal characteristics into a rich feature space, enhancing feature separability when combined with subsequent simple linear readout layer. The analogue switching behaviours adeptly emulate bioinspired synaptic operations that convey and process temporal information. For instance, the PPF effect allows the device to encode dynamic temporal input history of stimuli into nonlinear current responses, forming the basis for temporal mapping crucial in RC. To further enhance the temporal feature separability of the input signal, we introduce virtual nodes that extend the reservoir’s dimensionality by segmenting the time domain to form a high-dimensional representation. These virtual nodes capture the evolving internal states of the memristor and allow both past and present inputs to be embedded in the state trajectory of the system. Both past and present states of the time-series input can be encoded within a sequence of the internal states of memristors, where the conductance states of virtual nodes rely on historical data from physical nodes. As the number of virtual nodes increases, the required physical reservoir size of the recognition system can be further reduced, thereby minimising the hardware footprint without compromising system performance. Figure 4a conceptually schematizes the proposed RC paradigm employing the virtual-node concept. We demonstrate the feasibility of the PTB7-Th in-sensor RC system using a fingerprint recognition task. Given the established relationship between light intensity and the photocurrent of the memristor, we directly use the information from the original fingerprint images as the input of the reservoir layer to recognise the fingerprint with in-sensor RC. The images chosen from CASIA Fingerprint Image Database Version 5.0 (CASIA-FingerprintV5) are shown in Fig. S22. The 20 × 20 patterns, employing either binarized or analogue encoding of original image pixels, were converted into a spatiotemporal input by segmenting the pattern into 20 spatial inputs. Each spatial input was then applied to a distinct physical node, with each physical node comprising 20 virtual nodes. In the digital input scheme using a binarized dataset, each virtual node is composed of a 1 s pulse of 5 mV under dark conditions for a white pixel or 5 mV under light conditions for a black pixel. In the case of analogue input solutions, the memristors convert analogue dataset inputs into sequential voltage streams under light conditions, mapping the grey value [0 to 255] linearly to the voltage amplitude range [5 to 500 mV]. It is worth noting that the proposed RC system does not require an additional final reading pulse following the pulse stream to examine the changes in conductance, which can be attributed to the forming-free and analogue switching memristive operation. Figure 4b shows the responses to spike trains in two different channels taken from the same input as a representative. Although the similarities observed in the last part of the two input sequences, distinguishable differences emerge in the final virtual-node response. These differences are attributable to the earlier pulse sequences, highlighting the nonlinearity and temporal memory of the physical reservoir. The detailed pulse responses of all five fingerprint images under two distinct input schemes are included in Fig. S23. It is important to acknowledge potential variations in the initial conductance states among devices, despite efforts to maintain a consistent level. This variability is a merit of the RC system, as it allows for various states and even greater separability and adaptability.

We optimise the performance of the RC system by adjusting the number of parallel physical reservoirs (*M*) and virtual nodes (*N*), analysing the impact of each parameter change. To provide a comprehensive evaluation of the reservoir mapping performance, we use cross-entropy loss as a metric during the training process to quantify classification errors. Figure 4c shows the training loss for both digital and analogue schemes after 200 epochs varies with the diversification of the tunable node features, implying that the reservoir size (*M* × *N*) critically influences the performance. Expanding the reservoir by incorporating additional physical nodes (parallel memristors) and virtual nodes may lead to a further reduction in training loss, consequently enhancing classification accuracy. Nevertheless, this expansion increases hardware costs and training expenses for the subsequent readout layer. Additionally, the accuracy reaches 100% for all node features, with faster convergence for larger reservoir sizes, as shown in Figs. 4d and S24. The physical reservoir size can be further reduced as the number of virtual nodes increases. Insufficient virtual nodes may render the reservoir incapable of capturing key temporal features of the input signal, whereas an excessive number of virtual nodes may lead to a disregard for the significance of the signal correlation strength. Achieving optimal performance involves carefully tuning the RC to yield the lowest training loss in the digital or analogue schemes when *N* is approximately 5 (Fig. S25). We assess the biometric security system performance using the false rejection rate (*FRR*) and false acceptance rate (*FAR*). *FRR* is the percentage of identification instances in which authorised persons are incorrectly rejected, while *FAR* is the percentage of identification instances in which unauthorised persons are incorrectly accepted. Here we calculate the variation in *FRR* and *FAR* with the increasing training epochs as shown in Fig. 4e to better understand the accuracy of the proposed fingerprint recognition system. The *FRR* and *FAR* of 0% (100% recognition rate) were maintained after 100 epochs when *M* was set to 4 and 10, respectively, in the analogue configuration. The superior performance of the digital encoding scheme is due to its larger separable conductance states. However, it is still challenging to implement binary stimuli would require frequent toggling of illumination beneath the screen. By contrast, the analogue reservoir scheme bridge these gaps by combining with pre-processing functions of memristor-based sensor module, such as image contrast enhancement. Furthermore, the adaptability of the optoelectronic RC system is evident as it meets various requirements by accommodating various input conditions such as the electrical pulse amplitude and the illumination intensity.

### Fully memristive scheme for under-screen fingerprint recognition

Based on these crucial behaviours above, the same optoelectronic memristor can function as an optical sensor module, a physical reservoir module, and a readout module on demand, reducing the system complexity of edge devices. Utilising the unique advantages of this memristor, we can achieve a fully analogue, all-memristor optoelectronic RC architecture, serving as a versatile strategy applicable to various scenarios. As a proof of concept, we applied the analogue memristive optoelectronic reservoir scheme to an under-screen fingerprint recognition system, providing a low-power alternative approach. The under-screen optical fingerprint recognition system in smart devices places a sensor under the OLED display, sensing the brightness variations from the fingerprint unevenness to create an image. This image is compared by the central processing unit (CPU) and graphics processing unit (GPU) to the stored database. An additional optical collimator filter reduces background light, enhancing the image. While these systems are commercialised and energy-efficient, they require additional sensors and ADCs to convert optical analogue signals to digital inputs, making them less scalable and less affordable for future edge devices with limited battery access.

**Fig. 5:**
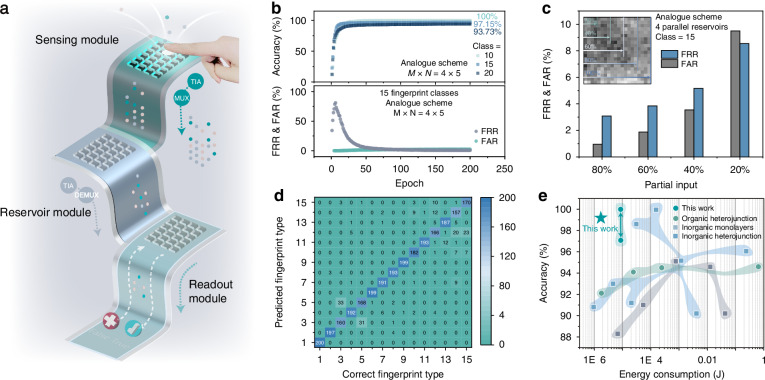
**Fingerprint recognition with a fully memristive in-sensor RC system.** **a** Schematic of the analogue scheme for under-screen fingerprint recognition. **b** Recognition accuracy of different fingerprint classes (top), *FRR* and *RAR* (bottom) of the 15 fingerprint recognition task, demonstrating the accurate classification results. **c** Low *FRR* and *FAR* shows the classification results obtained using 80%, 60%, 40%, and 20% of the input signal. Inset is the schematic of the classification using different portions of fingerprint data. **d** Confusion matrix illustrating the classification performance of the memristor-based reservoir system, using 20% of the input signal, in comparison with the correct output labels. Colour bar: occurrence of a given predicted output. **e** Comparison of previously reported optoelectronic memristor-based RC systems with our work in terms of system energy consumption and accuracy (see details in Table S2)

Figure 5a depicts the basic layout of the in-sensor analogue fingerprint recognition system, featuring under-screen PTB7-Th memristors in sensor, reservoir, and readout modes. In sensor mode, the memristor generates various photocurrents from reflected illumination signals, which are converted to voltage using a TIA and then pass through a MUX in reservoir mode. The output current of the reservoirs also feeds into the memristor-based readout module through TIA and MUX. The flexible light-transmitting film and the optical image taken with an inverted microscope (Fig. S26) demonstrate the potential of placing transparent memristor arrays under the screen. Note S2 and Fig. S27 demonstrate fingerprint optical sensing and signal pre-processing operations. The system performs in-sensor computing in the analogue domain via PTB7-Th memristors, realising diverse computational primitives from fingerprint sensing to recognition. To demonstrate our scalable network, we used 4 PTB7-Th memristor devices to experimentally implement the reservoir system with each device processing the analogue input spike train. The detailed current responses of the remaining 15 fingerprint image inputs are shown in Fig. S28. We selected five virtual nodes in the reservoir module. The output of the reservoir module is usually linearly separable, allowing for the training and classification of weights using a simple linear regression approach and a fully connected readout layer. Memristor devices with analogue linear updating conductivity (shown in Fig. S29) are designed for implementing fully connected readout networks^45^. Figure S30 showcases the stability and symmetry of memristor weights in response to 10 fixed optical and electrical pulses. The retention property of the optoelectronic memristor, as shown in Fig. S31, indicates that the trained weights from the local memristor array can be used for long-term recognition after offline training. These features are projected onto a fully connected readout layer with 20 sigmoid neurons at the last stage of RC. The noise-aware training method included Gaussian white noise with a standard deviation of 0.1 added to the experimental reservoir states, involving 800 training patterns and 200 testing patterns. For the 15-class fingerprint recognition task, our system achieves a high accuracy of 97.15%, underscoring its strong classification capability. As shown in Fig. 5b, the classification accuracy remains as high as 93.73% even when the number of fingerprint categories increases to 20, indicating the excellent scalability of the proposed approach. For the 15-class case, both the *FRR* and *FAR* remain low, demonstrating reliable identification performance. As illustrated in Fig. 5c, this robustness persists even when only a partial fraction of the input signals is used. The confusion matrix under the 20% input condition (Fig. 5d) further confirms that the 15 fingerprint classes are still clearly distinguishable, highlighting the effectiveness of the reservoir in handling sparse input data. The PTB7-Th memristor-based fully analogue in-sensor RC system has promising potential for achieving high-precision and robust fingerprint recognition tasks while simultaneously reducing network complexity. Developing and deploying emerging electronic devices with low-power and comparable performance to conventional devices remains challenging. Our design features an ultralow Energy consumption for high-accuracy reservoir computing, exhibiting significant advantages compared with previously reported reservoir electronic systems (Fig. 5e). The identical optoelectronic capabilities exhibited by 20 devices underscore the high yield of polymer memristors (Fig. S32). The device demonstrates consistent and promising stability even nearly 4 year after its fabrication (Fig. S33). The power consumption of the optoelectronic RC system is much lower owing to the low-power memristor and the simple architecture (see Note S3), and the operation energy can be further reduced by reducing the voltage pulse width, as shown in Fig. S34. Table S2 provides a detailed comparison with reported two-terminal optoelectronic memristive RC systems in terms of materials, structures, performance, and system energy consumption. Most reported works rely heavily on customised and complex material and structural design strategies to meet target memristor configuration demands, let alone the system complexity involved in integrating different structures on a monolithic circuit.

## Discussion

We proposed an in-sensor edge technology for analogue RC, leveraging a single type of optoelectronic polymer memristor. This technology enables dynamic voltage control of photocurrents, effectively integrating optical sensing, physical reservoir, and classification functions. The approach enables fingerprint recognition with 97.15% accuracy, using a compact reservoir and low system energy consumption. The forming-free and ultralow-voltage-driven system facilitates low-power training and inference operations, while fully analogue signal transmission further reduces computing latency. Additionally, the proposed device can be easily produced through a cost-effective and mass-scalable solution process. This technology promotes the adoption of organic polymer materials in mainstream integrated circuit manufacturing. The platform can be seamlessly integrated within an organic photovoltaic array, as it shares the same production process. This enables in-sensor computing for potential computation-on-chip, facilitating under-screen fingerprint recognition and other applications in future portable smart systems.

## Materials and methods

### Device fabrication processes

The ITO-coated glass substrates were cleaned with the cleaning agent and deionized water and baked at 120°C for 30 min in an oven. Thereafter, the substrates were UV-ozone treated for 1 h. The PTB7-Th solution was spin-coated (solvent chlorobenzene, 1500 r.p.m.) onto the substrates and then annealed at 110°C on a hotplate for 30 min in a glove box. Ag top electrodes (thickness of 25 nm) were thermally deposited onto the PTB7-Th film at a pressure of 5 × 10^−4^ Pa, resulting in a crossbar array with various cell areas. The silicon-based devices (p-type silica wafers), Au top electrode devices, and Cu top electrode devices were fabricated following the same procedures as described above.

### Electrical and optical measurements

Most electrical characterisations were conducted using a semiconductor parameter analyser (Keithley 4200s) inside a Cascade probe station in ambient air at room temperature. Electrical and optical measurements in a vacuum are performed using a Keithley 4200A-SCS. For the photoresponse measurements, 320 to 670 nm spectral output was supplied by a Xenon lamp by inserting a series of filters with adjustable power density. An optical power metre (Thorlabs PM100D) was used to calibrate the illumination intensity. The pulse optical signal was generated by using a mechanical shutter with controlled software. A comprehensive description of the readout layer training process is presented in Note S4.

### Structural and spectroscopic characterisations

The cross-sectional view of the PTB7-Th memristor was characterised by Hitachi S-4800 High Resolution SEM. The thickness of each layer was monitored by a Bruker Dektak XT stylus profiler. The morphologies and microstructures of the PTB7-Th films spin-coated on ITO and silicon substrates were investigated using Bruker’s Dimension Icon AFM. KPFM and c-AFM measurements were performed in tapping mode using Bruker’s Dimension Icon AFM with SCM-PIT and SCM-PIC type probe tips in ambient air, respectively. Absorbance measurements were performed using UV-vis spectroscopy (Perkin-Elmer Lambda 35). UPS data were collected on the XPS/UPS Photoelectron Spectrometer (*/KRATOS Axis Supra). Fluorescence emission spectra and fluorescence lifetimes were measured by Edinburgh FLS1000 Spectrometer. PL spectra were measured by an Edinburgh FLS920 spectrofluorometer using a white lamp as a light source. The TRPL data were obtained by FLS 980 Edinburgh Instruments. The 2D GIWAXS patterns were acquired using an XEUSS SAXS/WAXS system at the Brookhaven National Lab (New York).

## Supplementary information

Supplementary information for Optoelectronic polymer memristors with dynamic control for power-efficient in-sensor edge computing

## Data Availability

The data that support the findings of this study are available from the corresponding author upon reasonable request.
