# Supplementary material for: Optoelectronic polymer memristors with dynamic control for power-efficient in-sensor edge computing
Source: Light Sci Appl. 2025 Sep 8;14:309. doi: 10.1038/s41377-025-01986-9 (PMC12420826; doi:10.1038/s41377-025-01986-9)
Supplement: Supplementary file 1 — Supplementary information for Optoelectronic polymer memristors with dynamic control for power-efficient in-sensor edge computing [file 41377_2025_1986_MOESM1_ESM.pdf]

# Supplementary information for:

**Optoelectronic polymer memristors with dynamic control for power-efficient in-sensor edge computing**

Jia Zhou[1], Wen Li[1,*], Ye Chen[1], Haowen Qian[1], Yen-Hung Lin[2], Ruipeng Li[3], Zhen Wang[1], Jin Wang[1], Wei Shi[4], Xianwang Tao[4], Youtian Tao[4,*], Haifeng Ling[1], Wei Huang[1,4,*], and Mingdong Yi[1,*]

[1]State Key Laboratory of Flexible Electronics, Institute of Advanced Materials (IAM), Nanjing University of Posts & Telecommunications (NUPT), Nanjing 210023, China
E-mail: iammdyi@njupt.edu.cn; iamwli@njupt.edu.cn; vc@nwpu.edu.cn

[2]Department of Electronic and Computer Engineering, The Hong Kong University of Science and Technology, Clear Water Bay, Kowloon, Hong Kong SAR, China
State Key Laboratory of Advanced Displays and Optoelectronics Technologies, The Hong Kong University of Science and Technology, Clear Water Bay, Kowloon, Hong Kong SAR, China

[3]National Synchrotron Light Source II, Brookhaven National Laboratory, Upton, NY 11973, USA

[4]Key Lab for Flexible Electronics and Institute of Advanced Materials, Nanjing Tech University, Nanjing 211816, China
E-mail: iamyttao@njtech.edu.cn

These authors contributed equally: Jia Zhou, Wen Li

**Note S1. Mechanistic understanding**

Negligible contribution of the electrodes and interfaces by control experiments

Efficient memristive performance directly depends on a series of electronic processes that are closely influenced by structural and molecular factors. Next, we studied the underlying mechanism of the memristive property which reveals that homogeneous switching takes place across the entire polymer layer. The first step is to determine whether the optoelectronic memristive system in question is based on filamentary or non-filamentary processes, which is determined using cell-size-dependent electrical measurements. The pronounced dependence of resistance on the device area in Fig. 2a shows that switching is governed by spatially uniform interfaces. Zooming in from the 50 μm × 100 μm sample to the 500 μm × 100 μm sample reveals that the optoelectronic memristive behavior of the PTB7-Th devices is homogeneous on micrometer scales, with the photocurrent increasing as the device area increases. Different from the random distribution of conductive filaments in the filamentary conductance switching matrix, downscaling the PTB7-Th memristors to nanometer scale does not result in the spatial inhomogeneity of the defects and internal electric field while showing the stable optoelectronic properties in Fig. S1. The morphology and local optoelectronic resistive switching behavior of the PTB7-Th film, and its scalability into nanometer scales, were in situ monitored through conductive-atomic force microscope (AFM) analysis with a conductive-atomic force microscope (c-AFM) module. Exposure to white light increases the conducting capability of the polymer layer, and the entire image shows a remarkable and uniformly distributed current of more than 15 pA. This indicates that the scaling limit of the present polymer memristor can be extended reliably into the nanometer range. According to the formula $R = \rho L/S$, where $R$ is the resistance, $\rho$ is the resistivity, $L$ is the length, and $S$ is the active area of a resistive device, varying the polymer film thickness may result in different resistances of the memristors[1]. Consistent with this hypothesis, the devices with 80-nm-, 100-nm- and 120-nm-thick PTB7-Th film show similar and repeatable optoelectronic memristive characteristics in Fig. S2, while the photocurrent decreases as the polymer layer thickness increases. While the exact mechanism is still unknown, we measured the memristive characteristics of the PTB7-Th films with different electrodes and structures to rule out any electrode effects. Vertical structure devices with Au as the top electrode were fabricated, exhibiting the same memristive behavior in Fig. S3. The same phenomena were also exhibited in planar-structured devices in Fig. S4, which ruled out the influence of the interface on photoconductive performance. The currents of planar-structured memristors on the silica wafers also show a decrease with increasing channel lengths $L$. The above control experiments further reiterate that the photoresponsive

memristive behavior is intrinsic to the polymer film while being independent of the polymer layer size, thickness, and structure. In addition, devices that were not exposed to ambient air performed the same optoelectronic characteristics in a vacuum (Fig. S5). The results reveal that ambient exposure as well as the presence and absence of oxygen and moisture are not responsible for the optoelectronic memristive phenomena[2].

Exciton generation and dissociation processes

Having identified the essential roles of the polymer film, we propose that the operation of the optoelectronic memristor is enabled by the exciton generation process in the PTB7-Th film under light irradiation which is followed by charge transport and trapping processes. PTB7-Th is widely utilized as the donor material in organic solar cells to efficiently generate excitons, which dissociate to form geminate pairs. Donor-acceptor-type photoactive materials PTB7-Th integrate electron-rich monomers and electron-deficient monomers into one molecule. The photoexcited electrons were transferred from the donor unit to the acceptor unit under visible-light irradiation realizing an intramolecular electron transfer process[3], which can be revealed using X-ray photoelectron spectroscopy (XPS) analysis. The variation of S (2p) core-level spectra for PTB7-Th films under dark conditions and light exposure is presented in Fig. 2b. The S (2p) electron binding energy of thiophene in the BDT donor units is distinguished from that in the acceptor TT units according to the disparity in electron-richness of thiophene. The S (2p) electron binding energy in the donor part is denoted as $S^1$ and that in the acceptor part as $S^2$. After light exposure for 1 h, the $S^1$ (2p) peak moves to a higher binding energy while the $S^2$ (2p) peak moves in the opposite direction. This suggests that electron loss mainly occurs within the donor units and the electrons transfer to the acceptor units[4]. As shown in Fig. S6, the fluorescent emission peak intensity of PTB7-Th film in the room-temperature photoluminescence (PL) measurement gradually increases compared to the initial value after two minutes of light irradiation. Hence, it is reasonable to conclude that efficient exciton dissociation occurs in the PTB7-Th film as the exciton quenching in the aggregated states would decrease the emission intensity[5]. Correspondingly, the time-resolved photoluminescence (TR-PL) analysis of the PTB7-Th film shows that the intensity-weighted average carrier lifetime $\tau$ value increases affirming that the photogenerated excitons in PTB7-Th are easier transferred, and less recombined with the increasing light intensity (Fig. S7)[6]. Note that the XPS full spectrum and UV-vis spectrum of the PTB7-Th film provide further evidence for chemical stability at given experimental conditions as shown in Fig. S8. The

PTB7-Th film shows a strong absorption peak at 651 nm attributed to the $\pi$-$\pi^*$ transitions on the conjugated backbones while the shoulder peak at 711 nm is derived from the intermolecular interaction and order of the PTB7-Th molecule[7]. The UV spectrum before and after illumination hints at the stability of both the $\pi$-$\pi^*$ transitions on its conjugated backbones and its structural ordering. Moreover, it is reported that a crystalline form of organic semiconductor hampers exciton dissociation, and/or accelerates charge recombination[5,8]. AFM characterizations in Fig. S9 reveal a rather amorphous feature of PTB7-Th films as evidenced by the nearly featureless morphologies.

Charge transport and trapping processes

The charge transport process occurs after the injected charges first fill the traps. To directly verify the existence of trapped carriers in the film, Kelvin probe force microscopy (KPFM) was employed under light conditions with different light intensities to uncover the dynamic evolution of the potential distributions, as illustrated in Fig. 2c. It was observed that the contact potential difference ($V_{CPD}$) between the PTB7-Th film and the KPFM tip is lower at higher light intensities, which indicates an increase in the number of trapped holes. In Fig. S10, $V_{CPD}$ also increased significantly under light conditions and remained stable after light exposure. This indicates that positive charges would be generated under light conditions, which directly verifies the existence of trapped holes.

Physics-based transport models

To understand the underlying transport mechanism, we analyzed temperature-dependent $I$–$V$ characteristics using a thermionic emission framework. The extracted Arrhenius-type activation energy ($E$a ) reflects the energy required for carrier hopping over potential barriers. A lower Ea (Fig. 2e) for PTB7-Th on silicon, compared to ITO, indicates fewer deep traps and supports more efficient transport. Moreover, $E$a decreases with temperature, corresponding well with improved $I$–$V$ linearity at elevated temperatures (Fig. S11). The positive charges can continuously trigger electron injection after light illumination, thus leading to the Persistent photocurrent (PPC) behavior in the PTB7-Th film. When applying optical spikes with an energy exceeding the PTB7-Th energy bandgap (1.58 eV, as shown in Fig. S12), photoexcited excess electrons will be generated owing to the trapping of photogenerated holes, which results in a reduction in the work function of the PTB7-Th layer from 4.52 eV to 4.45 eV, confirmed via Ultraviolet Photoelectron Spectroscopy (UPS) measurements in Fig. S13. Simultaneously, the electron injection probability from the Ag electrode to the PTB7-Th layer is predicted to

increase, thereby increasing the conductance level. Here, the charge transport can be described by the thermionic emission[10], where a linear relationship is expected between $\ln(I/I_0)$ and $V^{1/4}$ as shown in Fig. S14. Most mobile carriers are electrons which contribute to the rise of photocurrent, while holes are localized immediately after being generated.

The photocurrent only exhibited a slight drop due to the carrier recombination after the removal of light. The holes trapped in the deep-level traps stably formed a space-charge layer, resulting in electron accumulation through a space-charge gating effect. The trapped holes can be slowly released due to the long lifetime thus the gating effect would exist for a very long time even after the illumination was terminated. As a result, the gating effect arising from trapped holes caused significant PPC behavior in the PTB7-Th devices. We can see that the photocurrent decay after light exposure follows a time-dependent bi-exponential decay, which implies the existence of a fast mechanism caused by intrinsic photoconduction and a slow decay mechanism caused by slow traps. Detailed analysis shows that the photocurrent decay curves can be well-fitted by a bi-exponential equation, given in Equation S1 below:

$$I = I_0 + A_1 \exp(-t/\tau_1) + A_2 \exp(-t/\tau_2) \quad \text{(S1)}$$

where $I_0$ is the photocurrent before the illumination is removed, $\tau_1$ and $\tau_2$ correspond to the time constants of fast and slow decay, respectively, and $A_1$ and $A_2$ are positive constants. It was reported that the fast decay component originates from the photoexcitation effect and the carrier fast recombination, while the slow decay component is due to the photogenerated effect caused by the deep traps[10]. Light intensity and light duration have little effect on the fast decay caused by intrinsic photoconductivity while increasing light intensity and light duration leads to more traps being filled, thus enhancing the slow decay process, as unambiguously verified by the quantitative analysis in Fig. S15.

### GIWAXS characterizations of PTB7-Th films and the control group

The photogenerated electron-hole pairs increase in the PTB7-Th layer under the light stimulus, while the holes are localized immediately, and the electrons contribute to the rise of photocurrent drift towards the electrode. Further information on the ordering of PTB7-Th films was obtained using GIWAXS in Fig. 2d. The high degree of disorder in the in-plane contributes to more traps for a greater chance of charge trapping and allows the exciton dissociation into free charges. The out-of-plane π-π stacking facilitates the charge transport from the active layer to the corresponding electrodes. We speculate that the defects brought about by the moderate structural disorder of the film can allow us to obtain the desired memristive performance, that is, the required linearity and nonlinearity, which can be confirmed by the film microstructure

and performance of the fabricated devices on the different substrates. Compared with the diffuse diffraction arc of the film on the ITO substrate, the film on the silicon substrate was observed to exhibit a more intense diffraction arc corresponding to the π-π stacking in the out-of-plane direction, which reduces energy disorder and defect states. Thus, clear linear conductance switching is shown in the *I-V* curves in Fig. S16a. However, the completely disordered state brings more defects and traps that hinder the achievement of ideal performance. Comparing the PTB7-Th films with different molecular weights, negligible differences regarding the molecular orientation or stacking modes can be observed[9]. However, it should be noted that the low molecular weight polymer sample (50 kDa) exhibits a broader arc-like scattering pattern indicating a more disordered structure. Since a higher degree of disorder promotes trapping during charge transport processes and induces more recombination losses, a negative effect on the stability of memristive performance as the degree of disorder increases is shown in Fig. S16b.

**Note S2. Pre-processing of fingerprint optical signals**

We use an 8 × 8 array consisting of PTB7-Th memristors where one memristor corresponds to one image pixel as an exemplary demonstration shown in Fig. S24. The human fingerprint images undergo cropping and resizing into 8 × 8 pixels to adapt to the light pulse input. By applying different light intensities corresponding to different light and dark fingerprint images to the given memristive pixels, the higher the light intensity, the higher the output current of the pixels. As a result, the fingerprint image is sensed and memorized in the array after removing the light source. The intensity-dependent photocurrent indicates that the memristor irradiated with a higher light intensity exhibits a longer retention time. As the photocurrent memory fades, the current differences between each pixel are enlarged over time, leading to a fingerprint image with enhanced contrast and highlighted key features, as well as removing low-intensity noisy points that might be introduced by contaminated objects or improper contact.

**Note S3. The estimated energy consumption of the PTB7-Th RC system in fingerprint recognition task**

We first calculated the average power consumption ($P_{avg}$) based on the electrical characteristics of the memristive devices in the system. In our analogue reservoir computing (RC) architecture, the input voltage and corresponding current vary within a limited range. For the fingerprint

recognition task, the input voltage pulses typically range from 0.005 V to 0.5 V, with corresponding measured currents in the range of 30–120 nA. Therefore, the $P_{avg}$ for processing a single input can be approximated as:

$P_{avg} = (0.005\ \text{V} \times 30\ \text{nA} + 0.5\ \text{V} \times 120\ \text{nA})/2 = 30.075\ \text{nW}$

Given an input pulse width of 130 ms (determined by hardware constraints in our measurement setup), the energy consumption per input is:

$E_{avg} = 30.075\ \text{nW} \times 130\ \text{ms} = 3.090975\ \text{nJ}$

In our fingerprint recognition task, we used 4 devices in parallel, corresponding to the optimal number of physical reservoirs ($M = 4$) required to achieve convergence in high recognition accuracy. Each device generates 20 virtual nodes, resulting in a total of 80 nodes per input. Therefore, the total electrical energy consumption for processing one input is:

$E_{Etotal} = E_{avg} \times 20 \times 4 = 3.090975\ \text{nJ} \times 80 = 312.78\ \text{nJ} \approx 0.313\ \mu\text{J}$

We also estimated the additional power and energy consumption induced by optical stimulation. The optical power $P_{light}$ is given by:

$P_{light} = P_d \times S$

where $P_d$ is the optical power density (5 mW cm$^{-2}$) and $S$ is the total active area of the 4 parallel physical reservoirs (100 μm × 100 μm × 4 = $4.0 \times 10^4$ μm$^2$ = $4.0 \times 10^{-4}$ cm$^2$). Thus, the total optical power is:

$P_{light} = 5\ \text{mW cm}^{-2} \times 4.0 \times 10^{-4}\ \text{cm}^2 = 2.0\ \mu\text{W}$

Assuming the optical pulse duration of 4 seconds (corresponding to the total time for 20 virtual nodes), the optical energy consumption is:

$E_{light} = P_{light} \times \text{t} = 2.0\ \mu\text{W} \times 4\ \text{s} = 8.0\ \mu\text{J}$

Hence, the overall energy consumption of our proposed optoelectronic RC system can be summarised as:

Total Energy Consumption = $E_{Etotal} + E_{light} = 0.313\ \mu\text{J} + 8\ \mu\text{J} = 8.313\ \mu\text{J}$

**Note S4. Network Simulations**

To emulate in-sensor reservoir computing with memristor-compatible architectures, we designed a two-layer readout neural network to decode the high-dimensional temporal features extracted by the optical reservoir. The input to the readout layer consisted of all the reservoir states with a feature size of $M \times N$ (where $M$ is the number of physical nodes and $N$ is the number of virtual nodes). The output neurons of the readout network corresponded to the fingerprint types. The Sigmoid and Softmax functions were used as activation functions for the hidden and output layers, respectively. The network was trained using a strategy that mimics

the physical behaviour of the memristive devices. Specifically, long-term potentiation (LTP) and depression (LTD) dynamics were first experimentally measured and fitted by the following phenomenological models:

$G_{\mathrm{LTP}}(\mathrm{n}) = G_{\mathrm{min}} + (G_{\mathrm{max}} - G_{\mathrm{min}}) \times (1 - \mathrm{e}^{-\alpha n})^{\beta}$

$G_{\mathrm{LTD}}(\mathrm{n}) = G_{\mathrm{max}} - (G_{\mathrm{max}} - G_{\mathrm{min}}) \times (1 - \mathrm{e}^{-\alpha n})^{\beta}$

where $G$ is the device conductance, $n$ is the number of applied optical pulses, and $\alpha$, $\beta$ are fitted parameters. The models enabled us to construct a lookup table linking the desired conductance $\Delta$G to the required number of potentiation or depression pulses.

During training, the loss function was defined as categorical cross-entropy, and the sparse categorical cross-entropy variant in TensorFlow Keras was used for computational efficiency. For benchmarking and comparison, a conventional training baseline using the Adam optimizer with a learning rate of 0.001 was implemented in simulation. However, in our physical training emulation, gradient descent was replaced with a pulse-driven weight update mechanism. Specifically, the gradient of the loss concerning each synaptic weight was computed, and the corresponding $\Delta G_{\mathrm{target}}$ was derived. The number of optical pulses needed to achieve this conductance change was then determined using the lookup table, and the weight was updated accordingly, bounded within the device's physical conductance window [$G_{\mathrm{min}}$, $G_{\mathrm{max}}$]. All weights were initialized within this accessible range, ensuring consistency with device behaviour. This physically-constrained, pulse-based training strategy directly incorporates memristor characteristics into the training loop, bridging the algorithm–hardware gap for in-sensor computing.

The accuracy of fingerprint recognition was evaluated as:

Accuracy(%) = (n/$N$) × 100%

where n is the number of correctly predicted samples and $N$ is the total number of test cases. In addition, the false rejection rate ($FRR$) and false acceptance rate ($FAR$) were calculated as:

$FRR = N_{\mathrm{FR}}/(N_{\mathrm{FR}}+N_{\mathrm{FA}}) \times 100\%$

$FAR = N_{\mathrm{FA}}/N_{\mathrm{IGA}} \times 100\%$

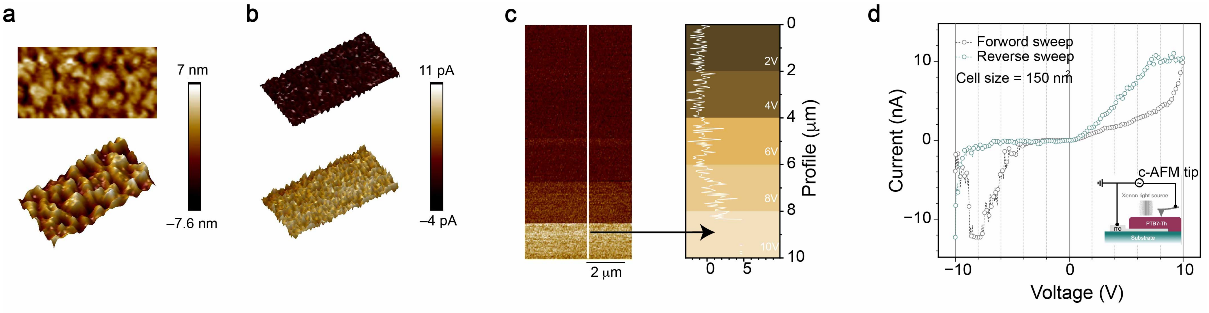


**Fig. S1 PTB7-Th film scalability into nanometer scales. a** Planar and 3D morphology of the PTB7-Th film. **b** The current mapping images of the film before and after white light exposure over the scanning area of 1.5 μm × 3 μm. **c** The morphology (left) and the corresponding current (right) undergo successively increasing voltage sweeps of the PTB7-Th film. **d** Memristive performance of the PTB7-Th film deposited on the ITO substrate where a c-AFM tip is used as the top electrode with a contact area of approximately 150 $nm^2$.

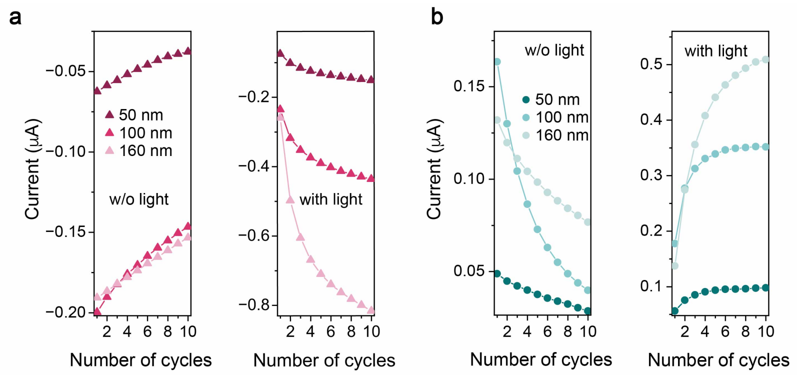


**Fig. S2 Thickness-dependent memristive behaviors of PTB7-Th devices.** Comparison of the memristive behavior under both (**a**) negative and (**b**) positive bias with polymer film thicknesses of 50, 100, and 160 nm, respectively. Light intensity: 5 mW $cm^{-2}$.

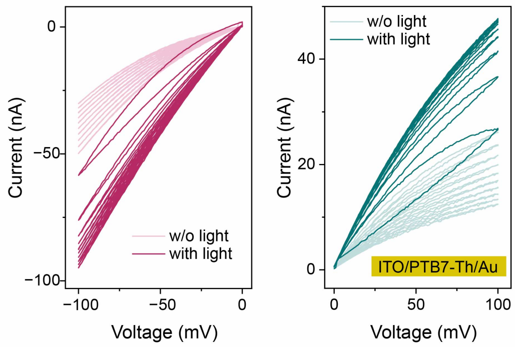


**Fig. S3 PTB7-Th device with Au top electrode.** The same memristive phenomenon is shown by the vertical PTB7-Th devices with Au top electrodes. Light intensity: 5 mW $cm^{-2}$.

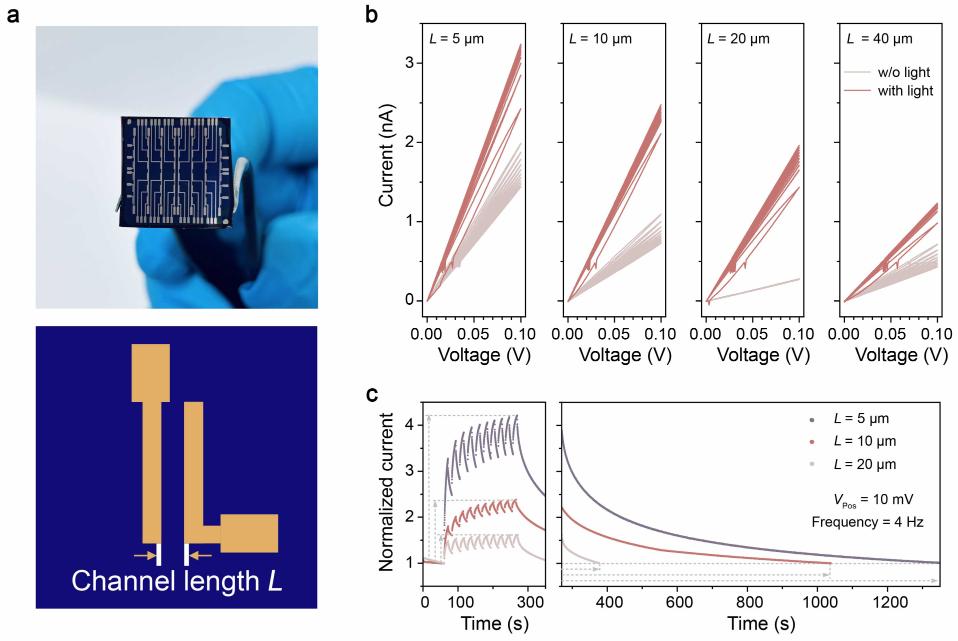


**Fig. S4 Memristive behaviors of planar-structured PTB7-Th devices. a** Optical image of the planar-structured PTB7-Th devices with different channel lengths (top) and the schematic image of the channel (bottom). **b** Memristive behaviors correspond to devices with different channel lengths *L* (planar structure: Au/PTB7-Th/Au). **c** The normalized photoresponse and the PPC effect of the planar-structured devices. Light intensity: 5 mW $cm^{-2}$.

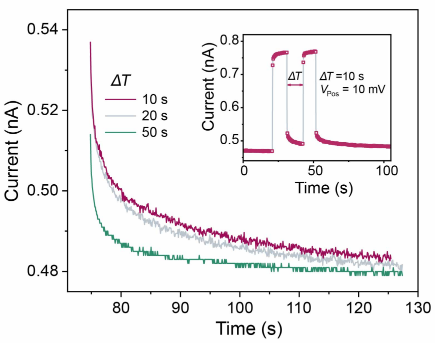


**Fig. S5 PPF ratio as a function of the optical pulse interval in vacuum.** Devices presented the same paired-pulse potentiation effect in a vacuum compared with the characteristics tested in ambient air, where paired optical pulses were applied (inset) with different intervals under 10 mV bias.

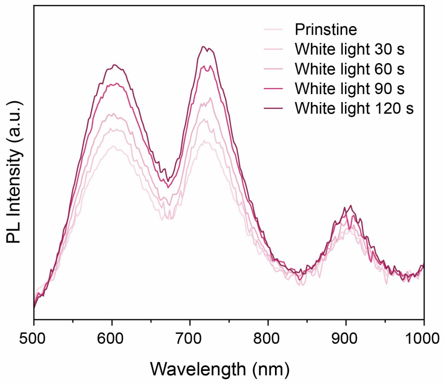


**Fig. S6 PL intensity evolution under different light durations.** The fluorescent emission peak intensity of the PTB7-Th film increases with the increase in light exposure durations.

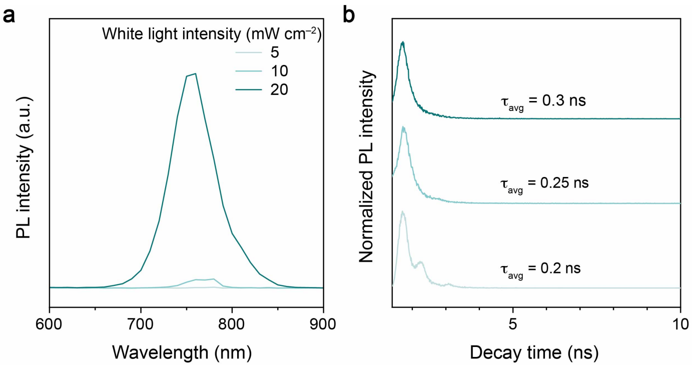


**Fig. S7 TR-PL analysis of PTB7-Th film. a** PL spectrum of the PTB7-Th film under light with different white light intensities. **b** TR-PL decay for the samples on the left half.

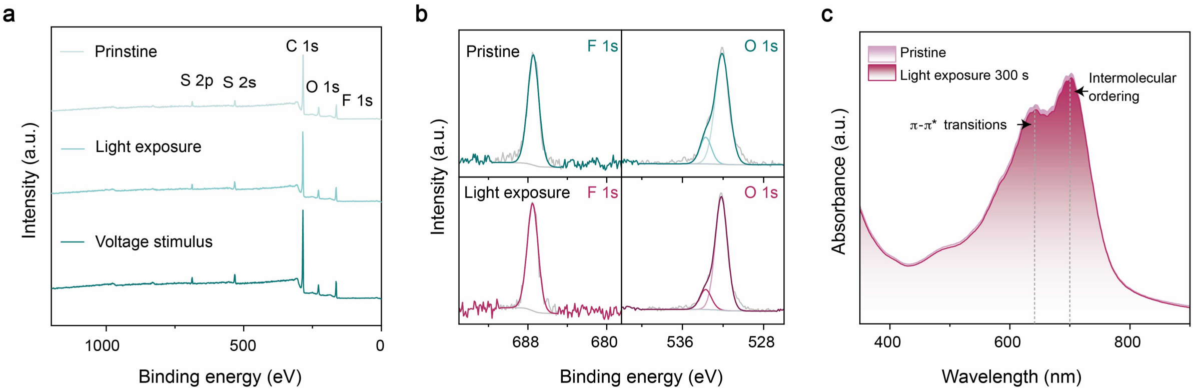


**Fig. S8 Chemical stability of PTB7-Th film confirmed by XPS and UV spectra. a** XPS full spectra of the as-prepared PTB7-Th layer on the ITO substrate. **b** Narrow scan of the F (1s) and O (1s) peaks before and after light exposure, respectively. **c** UV-visible spectra of the PTB7-Th film before and after light exposure.

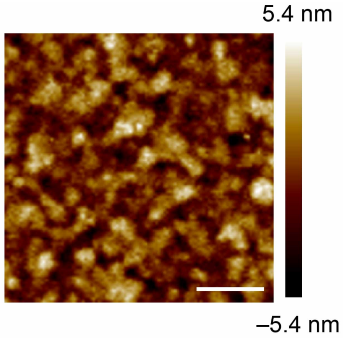


**Fig. S9 AFM image of the PTB7-Th film.** The image demonstrates its amorphous morphology (scale bar: 500 nm).

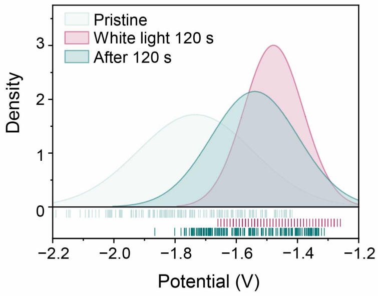


**Fig. S10 KPFM measurements of the PTB7-Th film surface potential before and after applying optical spikes.** The surface potential increased significantly by 164 mV after 120 s of light exposure and remained stable once the light was turned off.

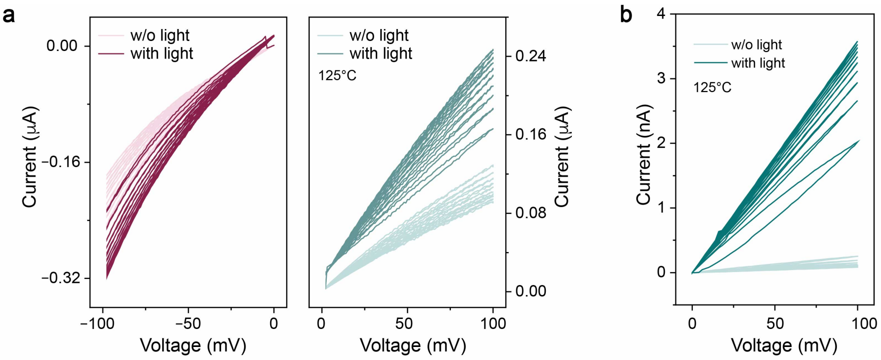


**Fig. S11 The repeatable *I*-*V* characteristics at high temperatures.** The (**a**) vertical and (**b**) planar-structured memristors at 125°C. Light intensity: 5 mW $cm^{-2}$.

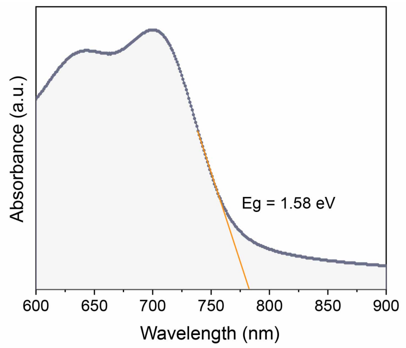


**Fig. S12 The absorption spectrum of PTB7-Th film on an ITO substrate.** The bandgap is extracted as 1.58 eV.

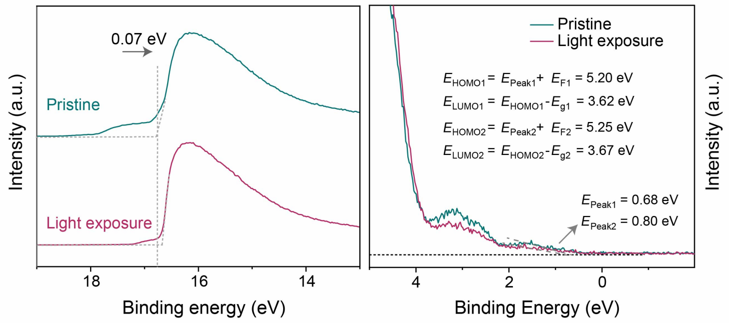


**Fig. S13 The energy level calculation of the PTB7-Th film.** UPS characterization of the PTB7-Th film on the ITO substrate with the cut-off region (left) and valence region (right).

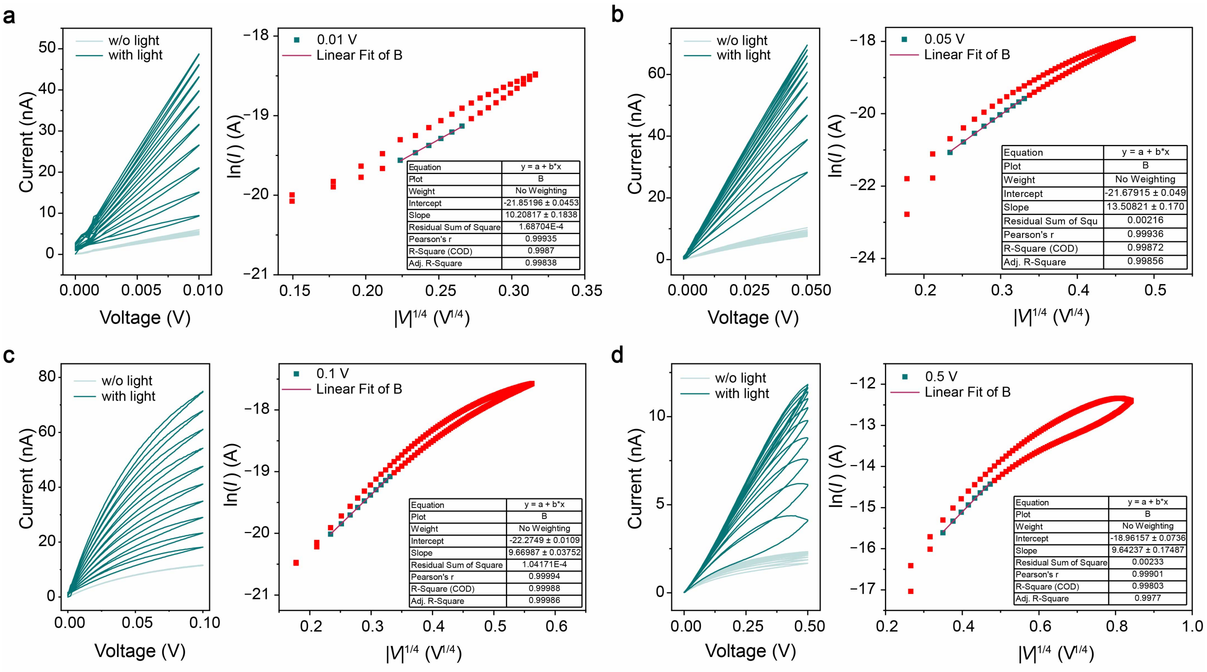


**Fig. S14 Transport characteristics of the PTB7-Th devices.** Plot of ln($I$) as a function of $|V|^{1/4}$ for the positive process in (**a**) 0.01 V, (**b**) 0.05 V, (**c**) 0.1 V, and (**d**) 0.5 V. The good linear

fit is consistent with the thermionic field emission model for charge injection. Light intensity: 5 mW cm$^{-2}$.

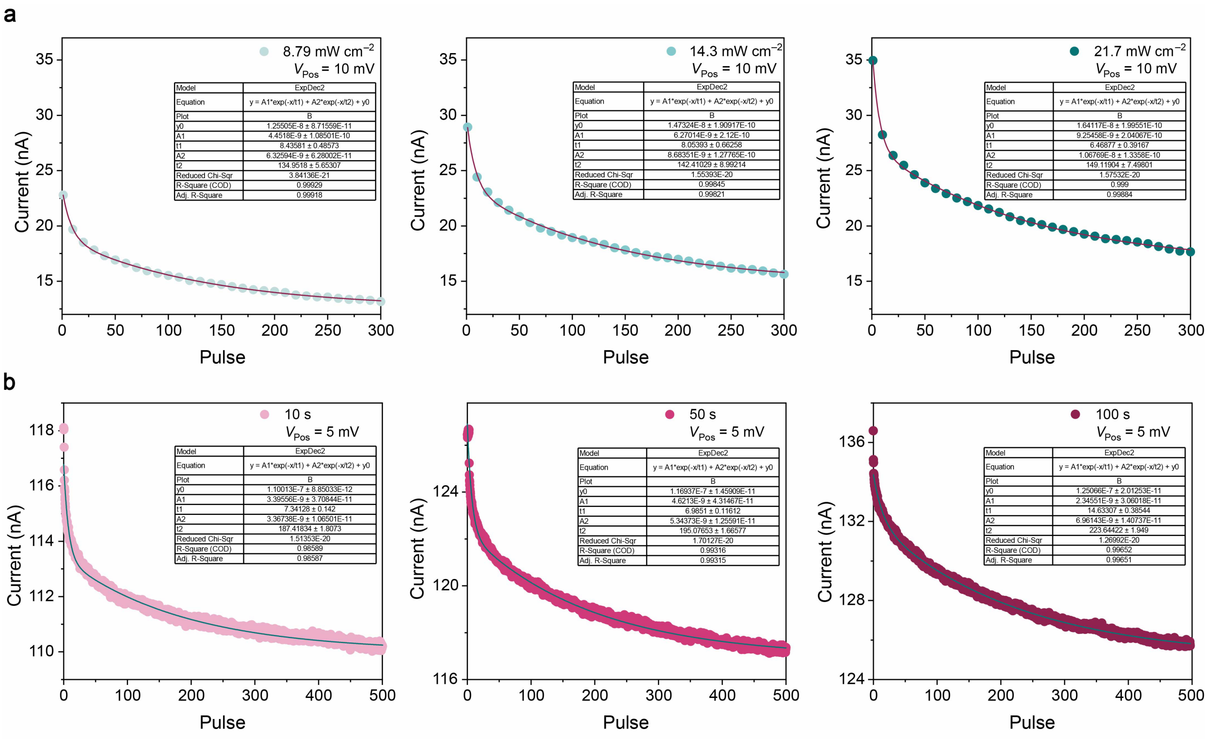


**Fig. S15 Fitting the photocurrent decay curves with a bi-exponential function:** $I = y_0 + A_1 \exp(-t/\tau_1) + A_2 \exp(-t/\tau_2)$. **a** The photocurrent response of the PTB7-Th memristor under light irradiation with different light intensities. **b** The photocurrent response of the PTB7-Th memristor under the same intensity of light irradiation with different light durations. The insets contain the fitting details of the corresponding photocurrent decay curves.

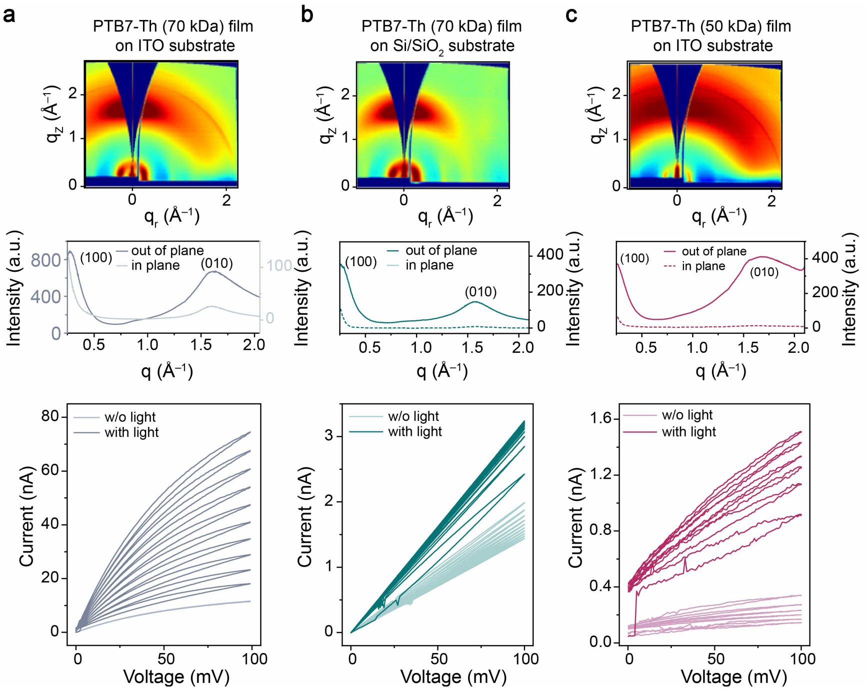


**Fig. S16 Structural properties analysed via GIWAXS and *I*-*V* characteristics of different control experiments. a**, PTB7-Th (molecular weight, 70 kDa) film on the ITO substrate. **b**, PTB7-Th (molecular weight, 70 kDa) film on the silicon substrate. **c**, PTB7-Th (molecular weight, 50 kDa) film on the ITO substrate.

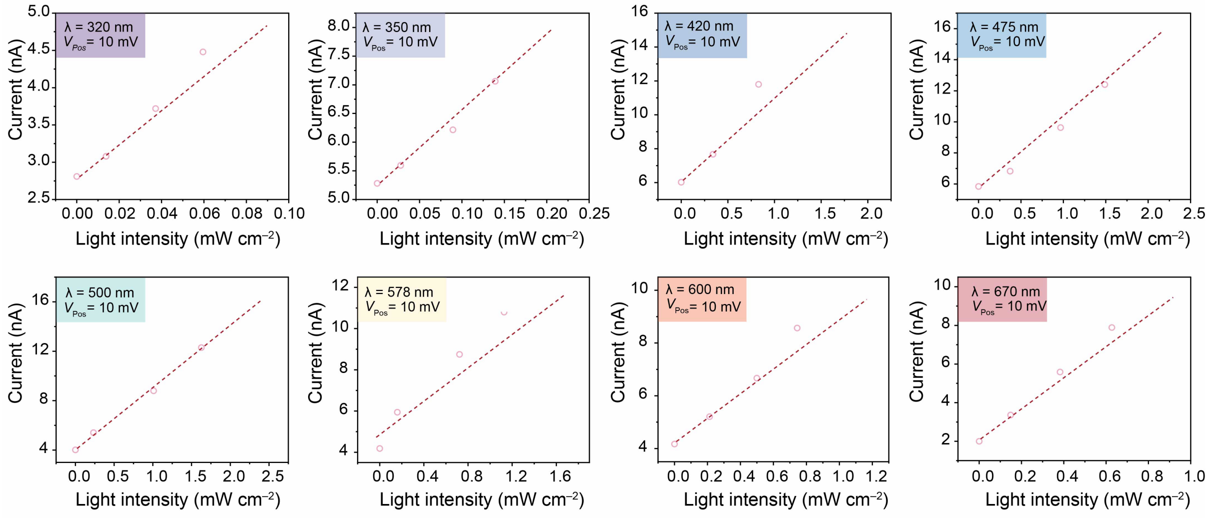


**Fig. S17 Broadband characteristics of the PTB7-Th optoelectronic memristor for in-sensor recognition.** The light intensity dependence of the extracted memristive photocurrent under eight different wavelengths of light illumination.

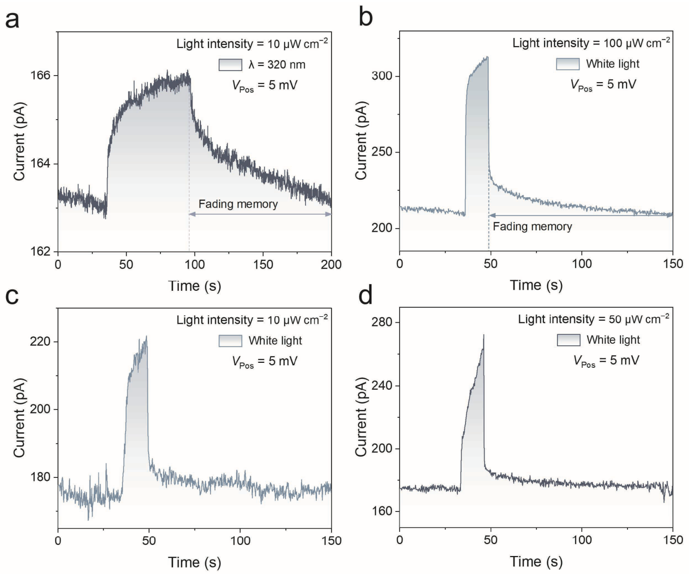


**Fig. S18** EPSC behaviour of the PTB7-Th device under **a** UV pulse with light intensity of 10 μW cm$^{-2}$ and under **b**–**d** white light pulses with various ultralow light intensities (10 to 100 μW cm$^{-2}$).

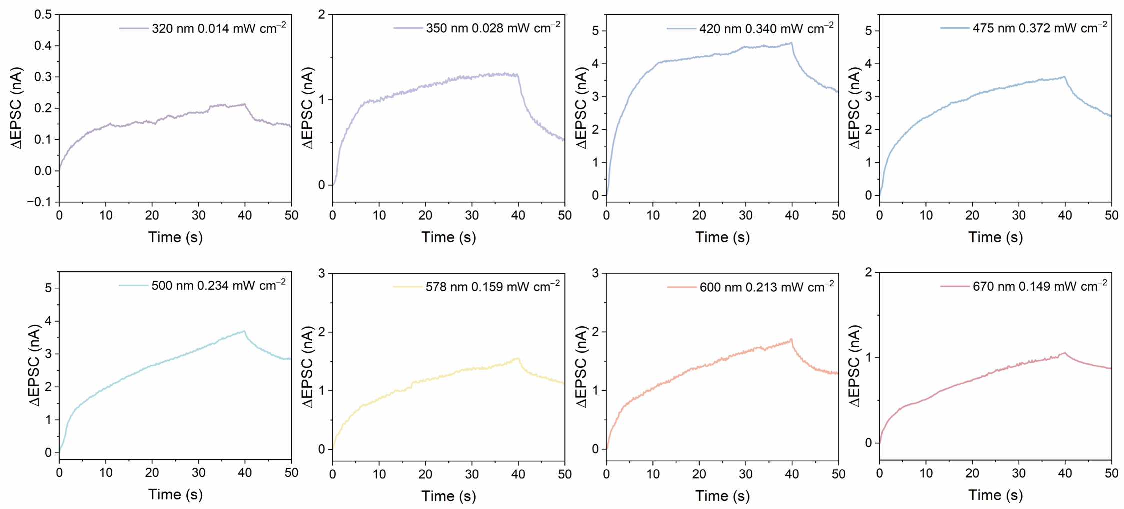


**Fig. S19** EPSC behaviour of the PTB7-Th device under different wavelengths (350–660 nm) with various ultralow light intensities.

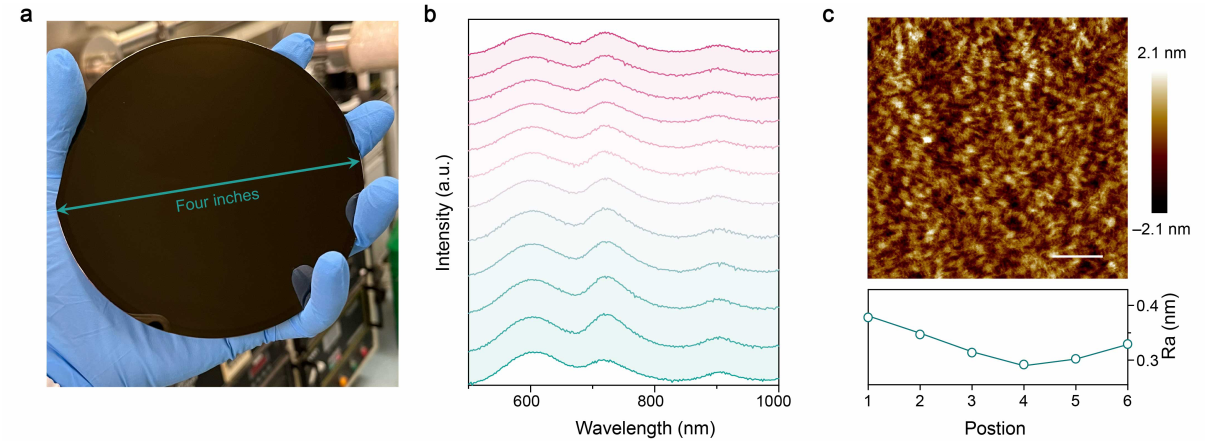


**Fig. S20 The PTB7-Th film for large-area electronics. a** PBT7-Th film homogeneously deposited on the four-inch wafer by solution process. **b** PL spectra at different positions of the four-inch wafer. **c** AFM topography (top) over the scanning area of 1 μm × 1 μm (scale bar: 200 nm) and the roughness distribution (bottom) of six randomly selected areas (10 μm × 10 μm) on the silica wafer.

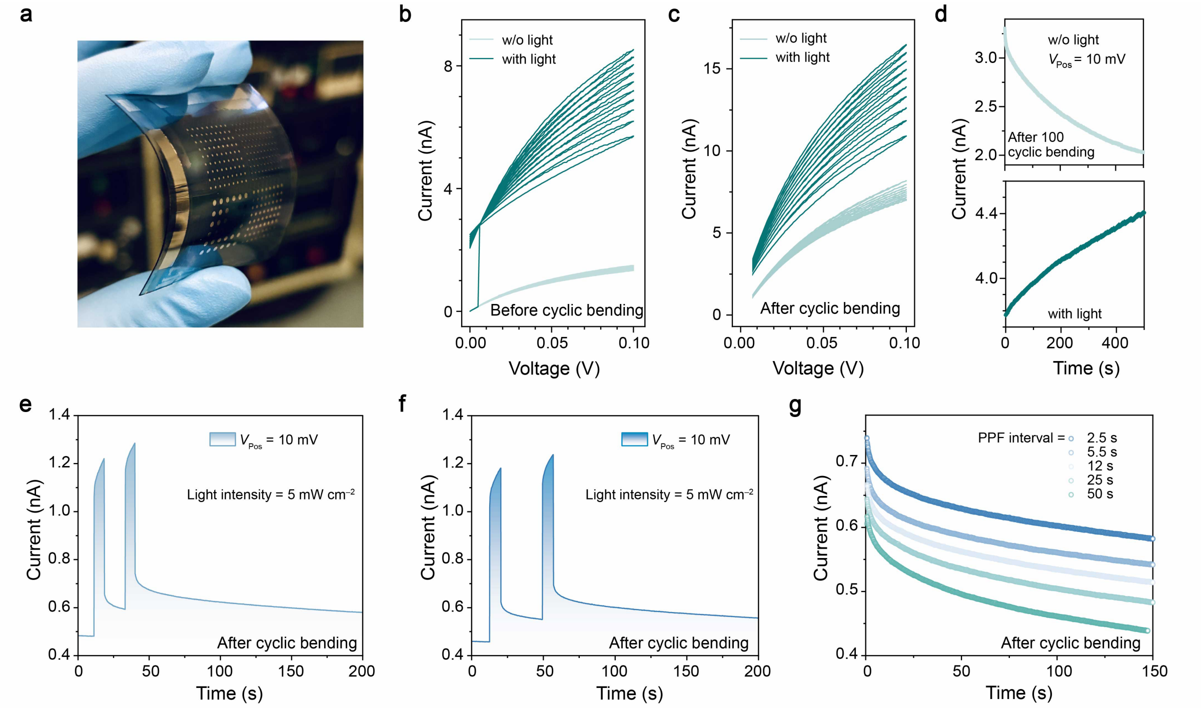


**Fig. S21 The optoelectronic memristive performance of a flexible PTB7-Th memristor.** The PTB7-Th film is introduced at each circular intersection with PET-ITO and Ag electrodes. **a** The optical image of the flexible devices in a bending state. **b** *I*-*V* characteristics of the flexible memristor in the flat state. **c** *I*-*V* characteristics and **d** optoelectronic performance of the flexible memristor after 100 cyclic bending. **e**–**f** PPF behaviors with different light pulse intervals. **g** The current decays after paired light pulses with different pulse intervals.

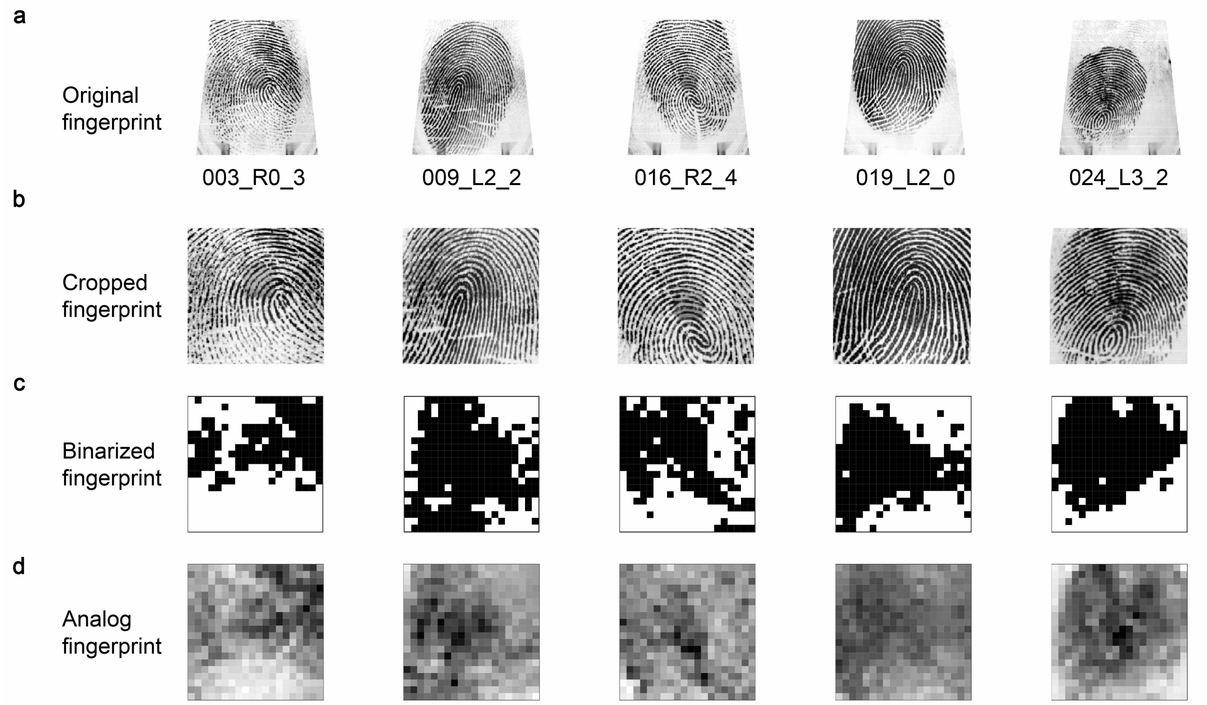


**Fig. S22 Overview of the five random fingerprint images from the CASIA-FingerprintV5 database utilized in this work.** **a** Five original fingerprint images. **b** The corresponding cropped fingerprint images. **c** The binarized fingerprint images. **d** The analog fingerprint images.

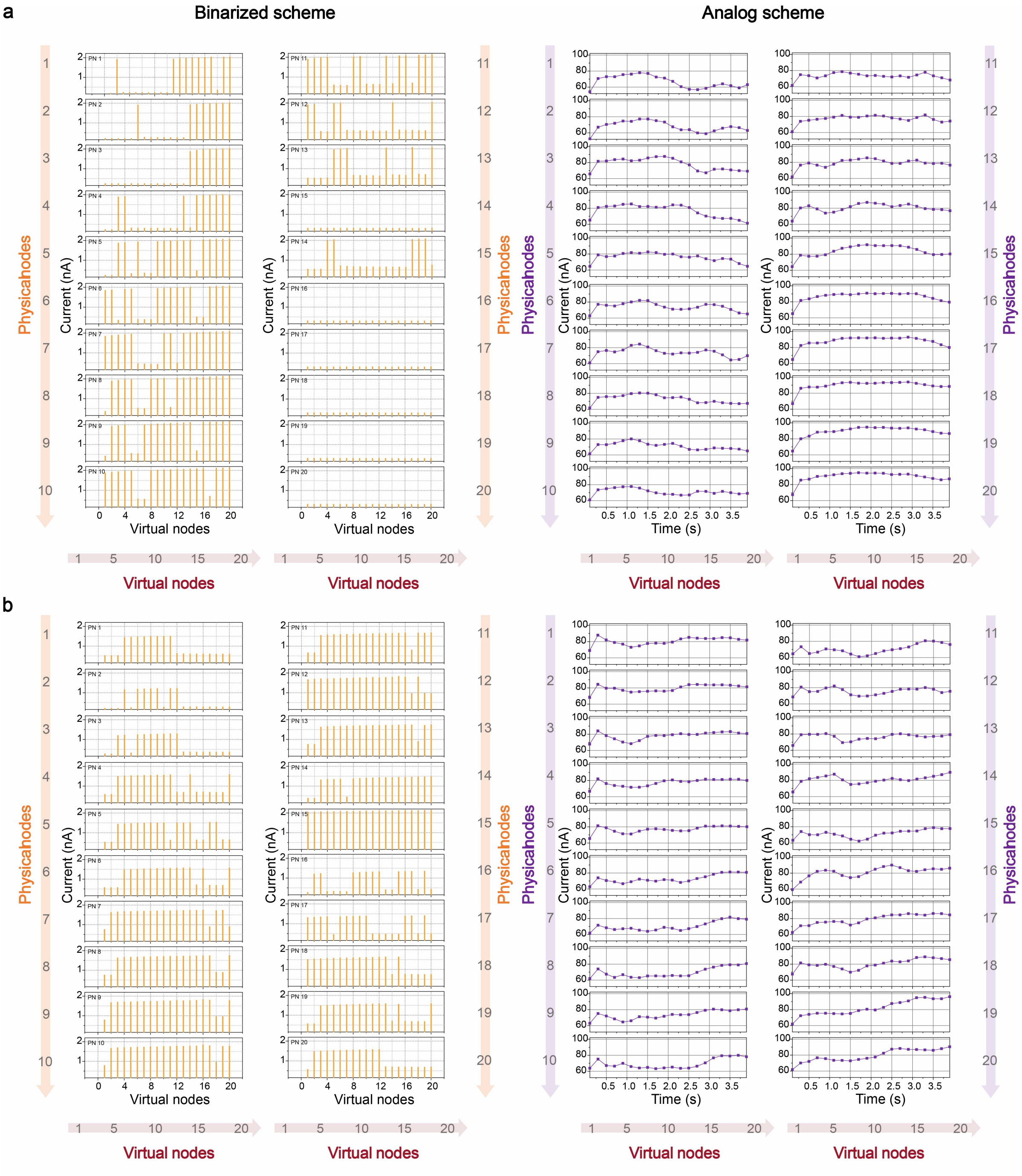
a
Binarized scheme
Analog scheme
Physicahodes
Current (nA)
Virtual nodes
Time (s)
Virtual nodes
b
Physicahodes
Current (nA)
Virtual nodes
Time (s)
Virtual nodes

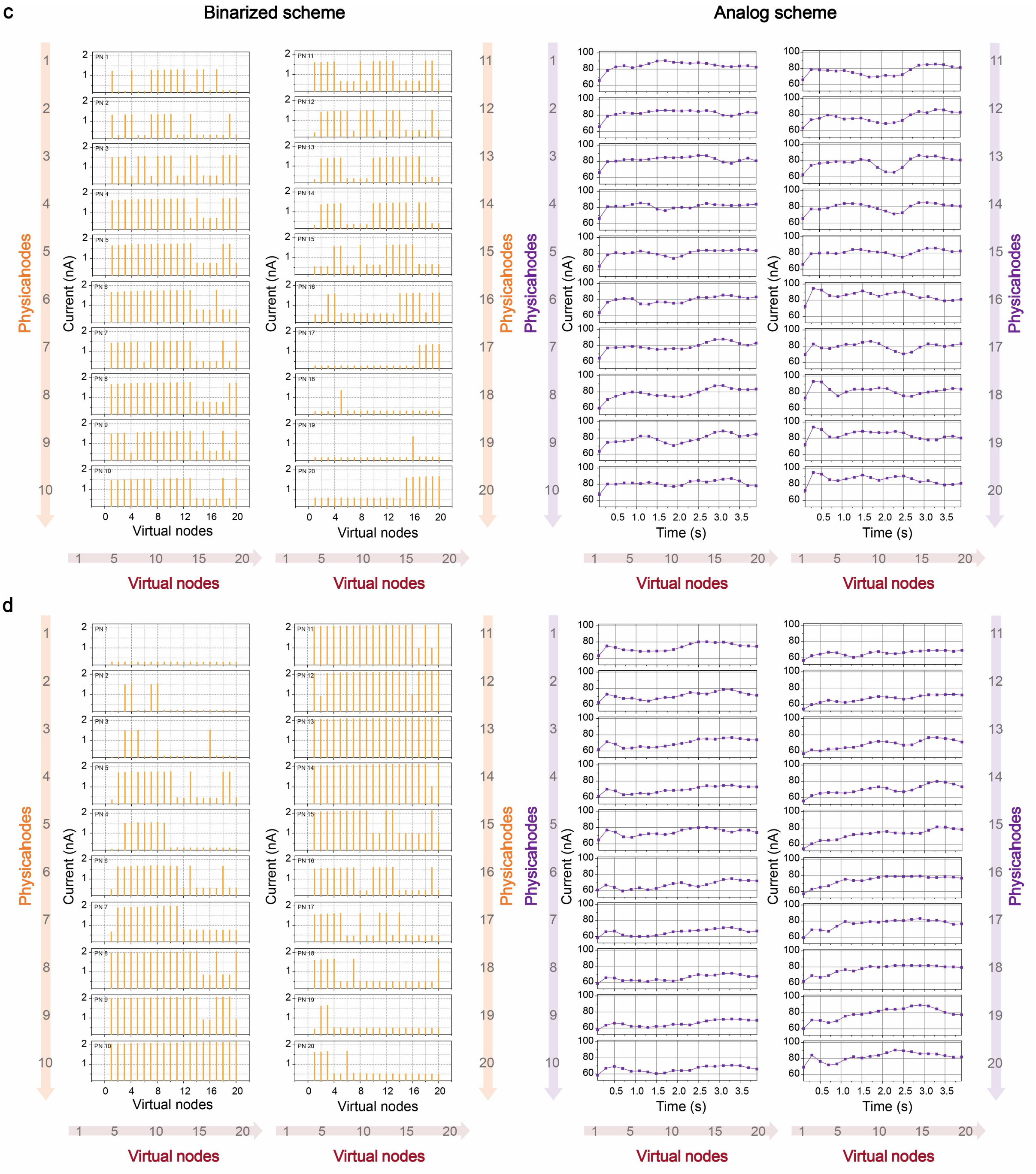
c
Binarized scheme
Analog scheme
d
Physicahodes
Current (nA)
Virtual nodes
Time (s)
PN 1
PN 2
PN 3
PN 4
PN 5
PN 6
PN 7
PN 8
PN 9
PN 10
PN 11
PN 12
PN 13
PN 14
PN 15
PN 16
PN 17
PN 18
PN 19
PN 20

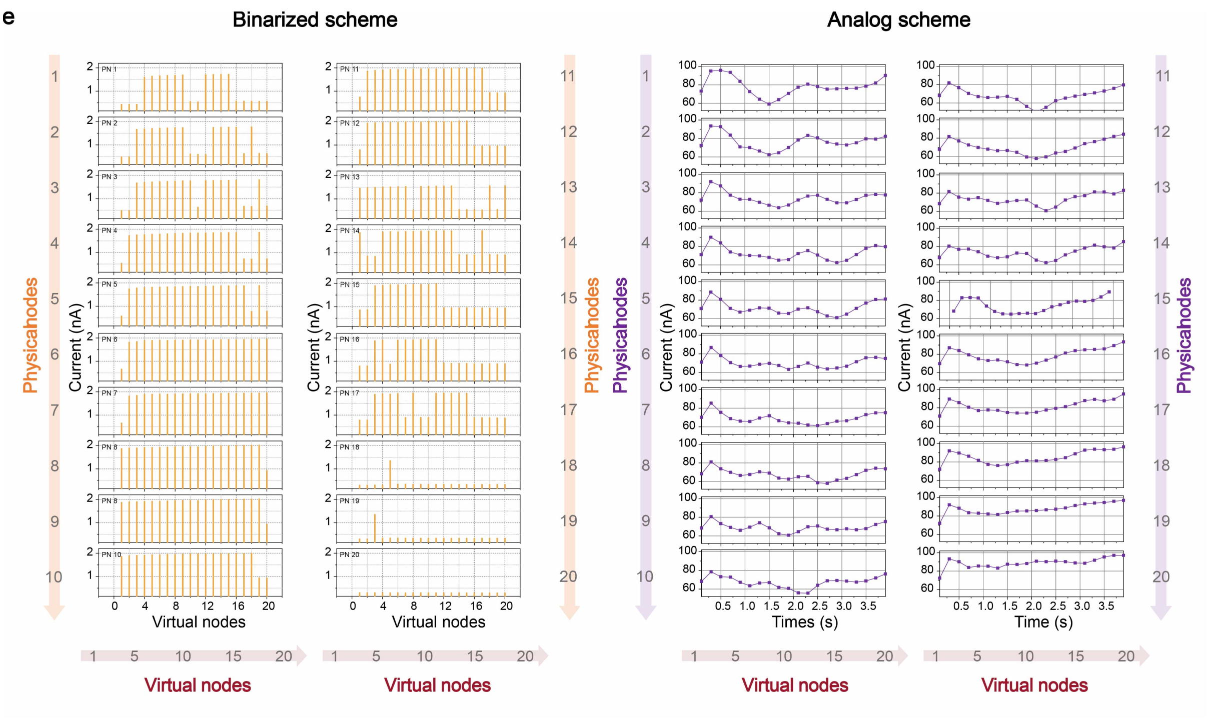


**Fig. S23 Detailed current responses of the fingerprint image inputs.** Experimental current responses were recorded under 20-pulse train stimulation using two encoding schemes of binarized (left) and analogue (right) based on five randomly selected fingerprint images. **a**–**e**, Fingerprint images 1–5. In the binarized scheme, each pixel is encoded into a 1 s, 5 mV pulse applied under dark conditions for a white pixel or under illuminated conditions for a black pixel. In contrast, the analogue scheme converts each grayscale pixel value (0–255) into a voltage pulse applied under illumination, with the amplitude linearly mapped to the range of 5 to 500 mV. These encoding methods generated sequential inputs for the readout network, enabling dynamic current response measurements across virtual nodes.

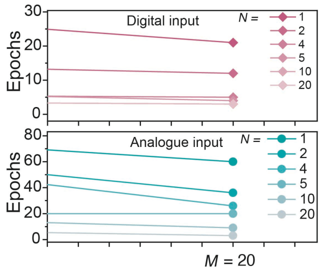


**Fig. S24** Magnified view of the number of training epochs required to achieve 100% recognition accuracy at different reservoir sizes.

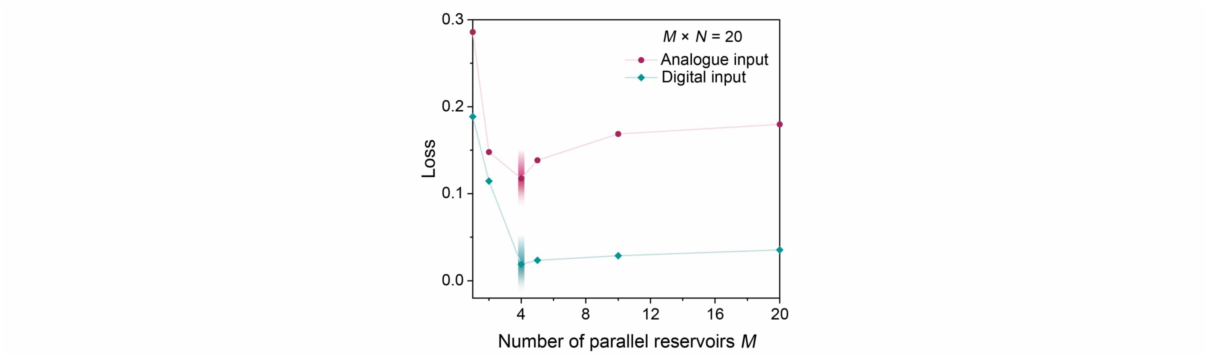


**Fig. S25 The reservoir performance varies with the number of virtual nodes *N*.** Training loss changes with the number of nodes when keeping the reservoir size (the number of physical nodes *M* and *N*) the same and the system achieves optimal performance as *M* reaches 4.

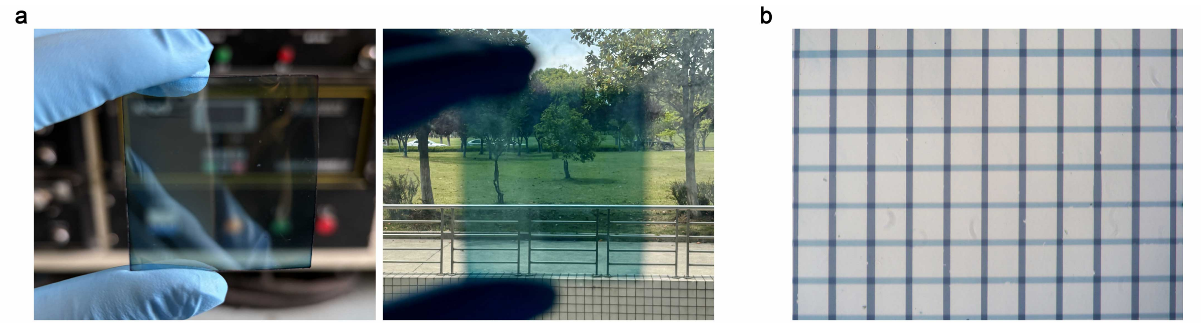


**Fig. S26 The light transmittance of the polymer film. a** The optical image of the uniformly transparent PTB7-Th film deposited on the PET substrate. **b** Optical image of the crossbar structure of the PTB7-Th memristor taken by an inverted microscope. The device size at each intersection point is 50 μm × 50 μm.

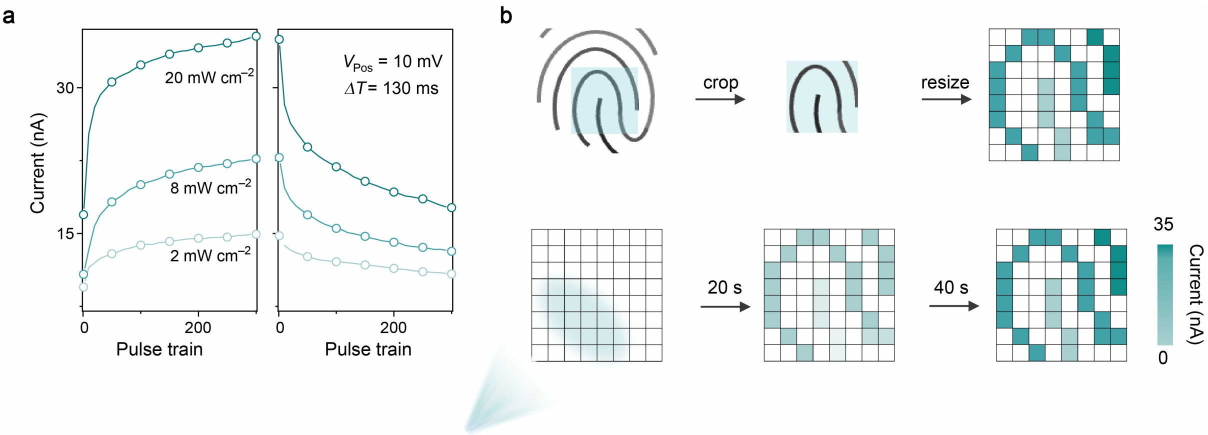


**Fig. S27 Fingerprint image pre-processing capability. a** The light intensity-dependent output currents, and retention time of the PTB7-Th memristor. **b** Signal sensing, memorizing, and contrast enhancement functions of the memristor array.

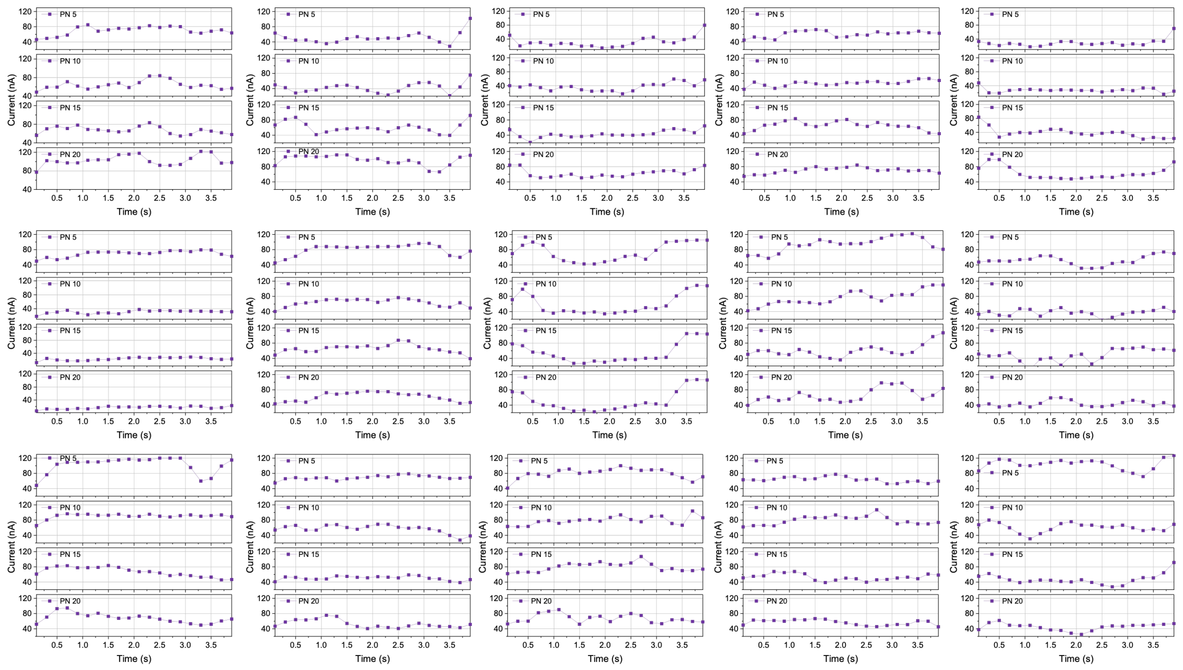


**Fig. S28** Detailed current responses to fingerprint image 6 to 20 inputs using the analogue encoding scheme, which converts each grayscale pixel value (0–255) into a voltage pulse applied under illumination, with the amplitude linearly mapped to the range of 5 to 500 mV. Reservoir size: 4 × 5.

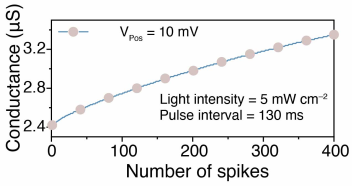


**Fig. S29** Linear conductance updating can be used for the weight updating and refreshing dynamics of readout layers.

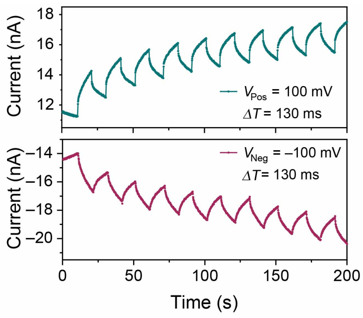


**Fig. S30 Symmetrically updated photocurrent of the PTB7-Th memristor**. The consecutive potentiation and depression characteristics triggered by optical stimulus read under relatively high applied voltage.

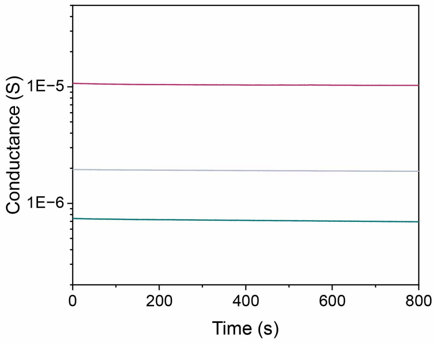


**Fig. S31 Retention measurement of multiple conductance states.** The conductance states were monitored for 800 s at each state and are stable.

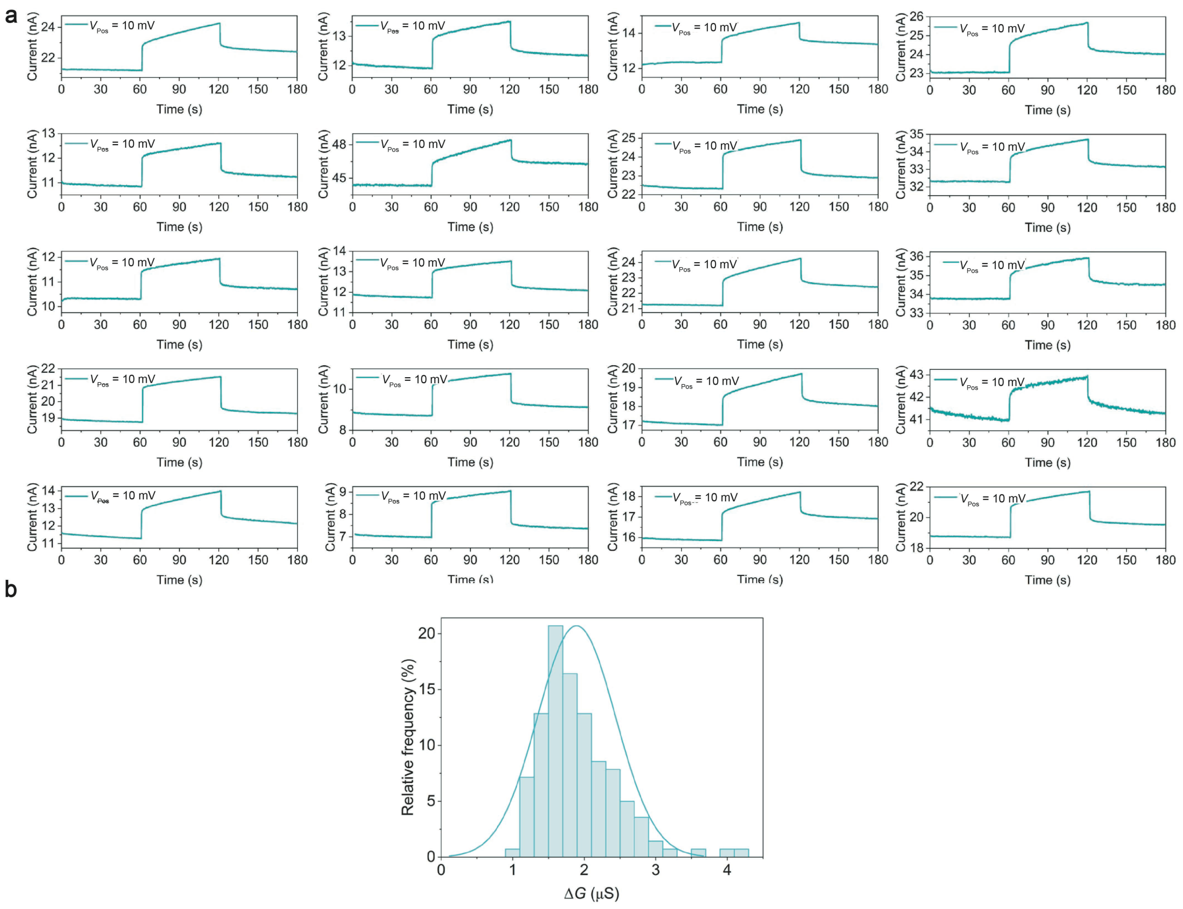


**Fig. S32 High yield production of PTB7-Th devices. a** Photoresponse of an array of 20 PTB7-Th Memristor devices, all curves obtained at a scanning voltage of 10 mV, with a white light intensity of 5 mW cm$^{-2}$ for 60 s. **b** Statistical distribution of conductance gain for the photoresponse of 20 PTB7-Th memristors under light for 60 s.

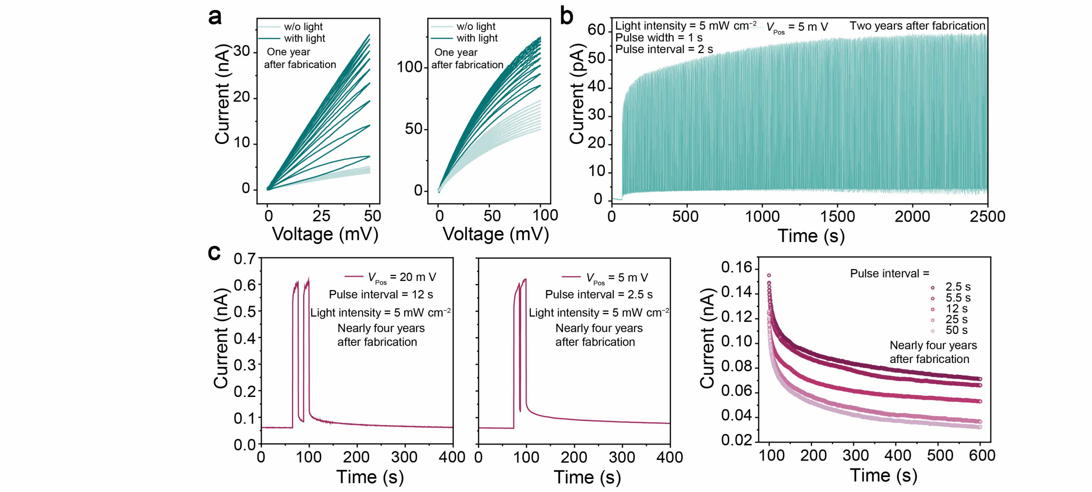


**Fig. S33 Air stability of the PTB7-Th devices. a** The stable memristive behaviors under different voltages are demonstrated one year after fabrication. Light intensity: 5 mW cm$^{-2}$. **b** Endurance test of the PTB7-Th memristor under consecutive light pulses two years after fabrication. **c** PPF behaviours of the PTB7-Th device nearly four years after fabrication.

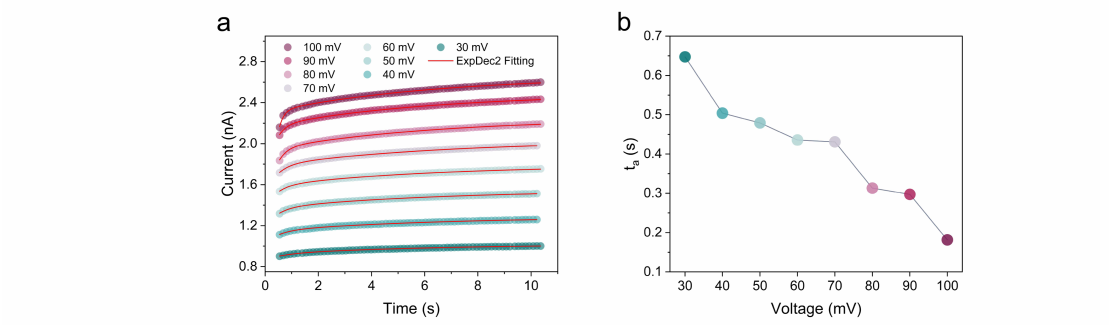


**Fig. S34 a** Photocurrent response of the PTB7-Th memristor under voltage pulses of varying amplitudes (30–100 mV). The device exhibits a rapid rise in current immediately upon voltage application, followed by a slower saturation phase, characteristic of dual-timescale dynamics. Follow the formula: $I = I_\infty - I_a \exp(-t/t_a) - I_b \exp(-t/t_b)$, Light intensity: 5 mW cm$^{-2}$. **b** Extracted fast characteristic time constant ($t_a$) as a function of pulse amplitude, obtained via double-exponential fitting of the data in Fig. S34a. $t_a$ decreases with increasing voltage.

**Table S1. Comparison of two-terminal organic memristors used in optoelectronic reservoir computing.** The 'V' and 'P' represent 'vertical-structured' and 'planar-structured' memristors, respectively.

| Materials | Structure | Device size [$\mu m^2$] | Array size | Wafer-scale | Wavelength [nm] | Intensity [$mW\ cm^{-2}$] | Task | Ref. |
|---|---|---|---|---|---|---|---|---|
| PTB7-Th | V | 50 × 50–500 × 500 | 20 × 20 | Y | 320–670 | 0.014–0.149 | Fingerprint recognition (CASIA-V5) | **This work** |
| C8-BTBT/CH-P | V | 100 × 100 | 8 × 8 | N | 450–630 | 5 | Falling behaviors recognition | [11] |
| Na:$MAPbBr_3$ | V | 80 × 80 | 5 × 5 | N | 300 | 0.21 | Dynamic image recognition (MNIST) | [12] |
| PEDOT:PSS /$CsCu_2I_3$ | V | 2000 × 100 | 22 × 3 | N | 375–445 | 11910 | Brain tumor detection | [13] |
| P3HT/GaAs | P | 1000 × 400 | 10 × 10 | N | 450–635 | 0.15 | Image classification | [14] |

**Table S2. Comparison of the state-of-the-art two-terminal optoelectronic memristive reservoir computing systems.** The ‘V’ and ‘P’ represent ‘vertical-structured’ and ‘planar-structured’ memristors, respectively. The ‘Y’ and ‘N’ stand for ‘Yes’ and ‘No’, respectively.

| | Materials | Structure | Task | Accuracy | Voltage | Current | Light intensity | System consumption | ADC /DAC | Flexibility | Ref. |
|---|---|---|---|---|---|---|---|---|---|---|---|
| | PTB7-Th | V | Fingerprint recognition (CASIA-V5) | 97.15% | 0.001–0.5 V | 30–120 nA | 5 mW $cm^{-2}$ | 8.31 μJ | N | Y | This work |
| | C8-BTBT/CH-P | V | Falling behaviors recognition | 94.1% | 0.05 V | 0.05 μA | 5 mW $cm^{-2}$ | 252.11 μJ | Y | N | 11 |
| Organic | Na:$MAPbBr_3$ | V | Dynamic image recognition (MNIST) | 92.11% | 0.4 V | 10.2 nA | 0.21 mW $cm^{-2}$ | 1.79 μJ | Y | N | 12 |
| | PEDOT:PSS /$CsCu_2I_3$ | V | Brain tumor detection | 94.6% | 0.05 V | 10 μA | 11.91 W $cm^{-2}$ | 0.64 J | Y | N | 13 |
| | P3HT/GaAs | P | Image classification | 94.5% | 15 V | 200 nA | 0.15 mW $cm^{-2}$ | 243.13μJ | Y | Y | 14 |
| | Nb-doped $SrTiO_3$ | V | Handwritten digit classification (MNIST) | 90.2% | 0.3 V | 190 nA | 5 mW $cm^{-2}$ | 4.13 mJ | Y | N | 15 |
| | IZO/$Al_2O_3$/TaN | V | Handwritten digit classification (MNIST) | 96.05% | 1 V | 405.5 μA | 2.1 mW $cm^{-2}$ | 0.24 J | Y | N | 16 |
| | N-CQDs | V | Handwritten digit classification (MNIST) | 94.58% | 0.2 V | 24 μA | 3 mW $cm^{-2}$ | 12.81 mJ | Y | N | 17 |
| | ZnO | V | Handwritten digit classification (MNIST) | 95.1% | 0.1 V | 1.55 μA | 0.5 mW $cm^{-2}$ | 765.18 μJ | Y | N | 18 |
| | ZnO/CdSe/ZnO | V | Image recognition (Fashion-MNIST) | 95.16% | 0.1 V | 70 μA | 6.1 mW $cm^{-2}$ | 1.19 mJ | Y | N | 19 |
| | ZnO/NiO | V | Letter image classification | 90.8% | 0.1 V | 200 pA | 38.2 μW $cm^{-2}$ | 0.946 μJ | Y | N | 20 |
| Inorganic | $TiO_x$/ZnO/TiN | V | Human motion recognition | 91.2% | 0.2 V | 7 μA | 10 mW $cm^{-2}$ | 20.63 μJ | N | N | 21 |
| | TiN/$TiO_2$/$NbO_x$/NiO | V | Image classification | 93.00% | 0.1 V | 30 pA | 4.51 mW $cm^{-2}$ | 4.62 μJ | Y | N | 22 |
| | $WO_{3-x}$/ZnMgO QDs/$WO_{3-x}$ | P | Image recognition(Fashion-MNIST) | 98.6% | 0.3 V | 53 nA | 66.2 μW $cm^{-2}$ | 30.90μJ | Y | N | 23 |
| | $WS_2$ | P | Handwritten digit classification (MNIST) | 88.3% | 0.1 V | 300 nA | 10 μW $cm^{-2}$ | 6.75 μJ | Y | N | 24 |
| | $V_3O_5$ | P | Handwritten digit classification (MNIST) | 90.2% | 0.5 V | 8 μA | 110 mW $cm^{-2}$ | 42.90 mJ | Y | N | 25 |
| | $MoS_2$/$CuInP_2S_6$ | P | Image classification | 99.95% | 0.1 V | 135 nA | 0.15 mW $cm^{-2}$ | 154.08 μJ | Y | N | 26 |
| | SnS | P | Sentences recognition | 91% | 0.5 V | 34 nA | 0.2 mW $cm^{-2}$ | 54.33 μJ | Y | N | 27 |

## References


1. Golfarelli, M., Maio, D. & Maltoni, D. On the error-reject trade-off in biometric verification systems. *IEEE Trans. Pattern Anal. Mach. Intell.* **19**, 786–796 (1997).
2. Dobryden, I. et al. Dynamic self-stabilization in the electronic and nanomechanical properties of an organic polymer semiconductor. *Nat. Commun.* **13**, 3076 (2022).
3. Qing, M. et al. Three-dimensional donor-acceptor-type photoactive material/conducting polyaniline hydrogel complex for sensitive photocathodic enzymatic bioanalysis. *Biosens. Bioelectron.* **158**, 112179 (2020).
4. Kang, H., Kim, K. H., Choi, J., Lee, C. & Kim, B. J. High-performance all-polymer solar cells based on face-on stacked polymer blends with low interfacial tension. *ACS Macro Lett.* **3**, 1009-1014 (2014).
5. Zhang, H. Q. et al. A centrosymmetric organic semiconductor with donor-acceptor interaction for highly photostable organic transistors. *Adv. Funct. Mater.* **32**, 2111705 (2022).
6. Hao, J. et al. Low-energy room-temperature optical switching in mixed-dimensionality nanoscale perovskite heterojunctions. *Sci. Adv.* **7**, eabf1959 (2021).
7. Chen, L. J., Wang, W., Xiao, S. Q. & Tang, X. F. Donor-acceptor conjugated copolymer with high thermoelectric performance: A case study of the oxidation process within chemical doping. *Chin. Phys. B* **31**, 028507 (2022).
8. Liu, J. et al. Relieving the photosensitivity of organic field-effect transistors. *Adv. Mater.* **32**, 1906122 (2020).
9. Hoefler, S. F. et al. The effect of polymer molecular weight on the performance of PTB7-Th:O-IDTBR non-fullerene organic solar cells. *J. Mater. Chem. A* **6**, 9506–9516 (2018).
10. Liu, K. Q. et al. An optoelectronic synapse based on alpha-$In_2Se_3$ with controllable temporal dynamics for multimode and multiscale reservoir computing. *Nat. Electron.* **5**, 761–773 (2022).
11. Lee, J. et al. Light-enhanced molecular polarity enabling multispectral color-cognitive memristor for neuromorphic visual system. *Nat. Commun.* **14**, 5775 (2023).
12. Wang, Y. et al. Sodium-enhanced perovskite reservoir for photonic in-sensor computing. *Nano Energy* **138**, 110830 (2025).
13. Zhang, L. L. et al. Lead-free halide perovskite-based optoelectronic synapse for reservoir computing. *Chem. Eng. J.* **506**, 160106 (2025).

14. Xie, P. S. et al. Birdlike broadband neuromorphic visual sensor arrays for fusion imaging. *Nat. Commun.* **15**, 8298 (2024).
15. Yamazaki, Y. & Kinoshita, K. Photonic Physical Reservoir Computing with Tunable Relaxation Time Constant. *Adv. Sci.* **11**, 2304804 (2024).
16. Park, H. et al. Long- and Short-Term Memory Characteristics Controlled by Electrical and Optical Stimulations in InZnO-Based Synaptic Device for Reservoir Computing. *Adv. Electron. Mater.* **10**, 2300911 (2024).
17. Yu, T. Q. et al. Integrated in-memory sensor and computing of artificial vision system based on reversible bonding transition-induced nitrogen-doped carbon quantum dots (N-CQDs). *Nano Res.* **17**, 10049-10057 (2024).
18. Jiang, J. D. et al. Hardware-Level Image Recognition System Based on ZnO Photo-Synapse Array with the Self-Denoising Function. *Adv. Funct. Mater.* **34**, 2313507 (2024).
19. Lin, Q. H. et al. A Full-Quantum-Dot Optoelectronic Memristor for In-Sensor Reservoir Computing System with Integrated Functions. *Adv. Funct. Mater.*, 2423548 (2025).
20. Fang, Y. Q. et al. Photonic Synapses for Image Recognition and High Density Integration of Simplified Artificial Neural Networks. *Adv. Electron. Mater.* **9**, 2300120 (2023).
21. Huang, H. Y. et al. Fully integrated multi-mode optoelectronic memristor array for diversified in-sensor computing. *Nat. Nanotechnol.* **20**, 93–103 (2025).
22. Lu, C. et al. Reconfigurable Selector-Free All-Optical Controlled Neuromorphic Memristor for In-Memory Sensing and Reservoir Computing. *ACS Nano* **18**, 29715-29723 (2024).
23. Guo, Z. L., Kan, H., Zhang, J. Q. & Li, Y. Neuromorphic Visual Computing with ZnMgO QDs-Based UV-Responsive Optoelectronic Synaptic Devices for Image Encryption and Recognition. *Small* **21**, 2412531 (2025).
24. Gong, Y. et al. Integrated Bionic Human Retina Process and In-Sensor RC System Based on 2D Retinomorphic Memristor Array. *Adv. Funct. Mater.* **34**, 2406547 (2024).
25. Nath, S. K. et al. Optically Tunable Electrical Oscillations in Oxide-Based Memristors for Neuromorphic Computing. *Adv. Mater.* **36**, 2400904 (2024).
26. Xia, Y. P. et al. 2D Reconfigurable Memory Device Enabled by Defect Engineering for Multifunctional Neuromorphic Computing. *Adv. Mater.* **36**, 2403785 (2024).

27. Sun, L. F. et al. In-sensor reservoir computing for language learning via two-dimensional memristors. *Sci. Adv.* **7**, eabg1455 (2021).
